# Identification of early metabolic indicator candidates in scion leaves associated with delayed incompatibility in tomato and pepper grafting via metabolism

**DOI:** 10.3389/fpls.2026.1919357

**Published:** 2026-09-01

**Authors:** Ni Chen, Fanshu Sang, Heng Liu, Shuang Li, Dongliang Li, Siyuan Wu, Junjie Guo, Jiachen Liu, Jingjing Chen

**Affiliations:** 1 School of Life and Environmental Sciences, Shaoxing University, Zhejiang, China; 2 South Subtropical Crops Research Institute, Chinese Academy of Tropical Agricultural Sciences; Key Laboratory of Tropical Fruit Biology, Ministry of Agriculture & Rural Affairs, Zhanjiang, Guangdong, China; 3 College of Life Sciences, Sichuan Normal University, Chengdu, Sichuan, China

**Keywords:** graft, incompatibility, metabolic indicator candidates, pepper, tomato, metabolomics, scion leaves, biomarkers

## Abstract

**Introduction:**

The delayed grafting incompatibility leads to a time-consuming, expensive, and labor-intensive agricultural process for the evaluation and transfer of new varieties to the industry. Current diagnostic approaches predominantly rely on sampling from the graft interface, which is invasive and may disrupt normal plant development. Identifying metabolic indicators in scion leaves offers a non-invasive alternative with minimal interference to grafted plants. However, reports on metabolic biomarkers of graft incompatibility in scion leaves remain limited, particularly for the tomato (*Solanum lycopersicum* cv. Zhongshu No. 4) and pepper (*Capsicum annuum* cv. Hangjiao No. 1) grafting combination. This study aimed to identify early metabolic indicator candidates in scion leaves for the detection of delayed graft incompatibility using a widely targeted metabolomics approach.

**Methods:**

Tomato/tomato (TT, compatible self-grafting) and tomato/pepper (TP, delayed incompatible heterografting) combinations were established. The fourth true leaf of tomato scions was collected at 5 days after grafting (DAG) for metabolomic analysis. Metabolites were extracted and analyzed using ultra-performance liquid chromatography-tandem mass spectrometry (UPLC-MS/MS). Multivariate statistical analyses, including principal component analysis (PCA), orthogonal partial least squares discriminant analysis (OPLS-DA), and hierarchical cluster analysis (HCA), were performed to identify differential metabolites. Metabolic pathway enrichment was analyzed using the KEGG database and metabolite set enrichment analysis (MSEA). Differential metabolites were screened based on VIP > 1, |Log2FC| ≥ 1.0, and Student’s t-test p < 0.05 with Benjamini-Hochberg FDR correction. Exogenous validation experiments were conducted by treating TT-compatible grafts with selected metabolites to assess their effects on graft survival.

**Results:**

A total of 207 differential metabolites were identified between TT and TP scion leaves, comprising 68 primary metabolites and 139 secondary metabolites. Metabolic pathway enrichment analysis revealed eight significantly enriched pathways. Among these, 31 differential metabolites were further characterized, including 24 primary metabolites and 7 secondary metabolites. Specifically, 23 metabolites were up-regulated and 8 were down-regulated in TP. The up-regulated metabolites were predominantly saccharides, whereas the down-regulated metabolites were mainly associated with flavonoid biosynthesis. Exogenous application of four primary metabolites (glucose, sucrose, mannose, trehalose) and one secondary metabolite (rhoifolin) significantly reduced the survival rate of TT-compatible grafting, confirming their adverse effects on graft compatibility.

**Discussion:**

These findings demonstrate that scion leaves can serve as a viable, non-invasive material for identifying potential biomarker candidates of delayed graft incompatibility, offering comparable diagnostic value to graft interface sampling but with greater convenience. Metabolites identified in scion leaves exhibit significant effects on graft compatibility and may function as early indicators of incompatibility. Furthermore, pathway-level metabolic analysis provides more comprehensive diagnostic insights than single-metabolite profiling, suggesting that a systems-level approach to metabolic characterization is essential for understanding graft incompatibility mechanisms. Future studies should validate these metabolic signatures across multiple cultivars and time points to establish their predictive utility.

## 1 Introduction

Plant grafting, as an ancient and widely utilized agricultural technique, plays an indispensable role in the conservation of germplasm resources, improvement of agronomic traits, control of agricultural pests and diseases, and the breeding of new varieties (especially useful rootstocks) (Warschefsky et al., 2016; Gaion et al., 2018; Žanić et al., 2018; Williams et al., 2021). However, for a specific crop, the selection of compatible rootstocks in practical is typically restricted (Tedesco et al., 2022). Grafting compatibility generally decreases between different species within the same genus and becomes even more incompatible between different genera within the same family. Although an important study has demonstrated that *Nicotiana benthamiana* exhibits grafting compatibility with numerous species across different families and GH9B3 (β-1,4-glucanase) proved to be a key factor (Notaguchi et al., 2020), incompatible grafting remains largely unpredictable.

Graft incompatibility refers to the phenomenon wherein the complex intrinsic genetic interactions between the rootstock and the scion impair the wound-healing process, hinder the regeneration of vascular tissues, and obstruct the reconstruction of the vascular system. This ultimately results in the death of the grafted plant or disorders in its growth and development (Notaguchi et al., 2020; Thomas et al., 2021; Tedesco et al., 2022; Pina et al., 2021; Habibi et al., 2022; Babar et al., 2023).

The grafting process involves a sophisticated genetic regulatory network, encompassing aspects such as the activation of wound response mechanisms, the induction of oxidative stress and secondary metabolites, the interaction of multiple hormonal signals, and the expression of regeneration-associated genes (Yin et al., 2012; Melnyk et al., 2015; Nanda and Melnyk, 2018; Feng et al., 2024). From the perspective of morphogenesis, the graft healing process can be classified into four key stages: (1) the wound response and cell wall modification at the graft interface; (2) alterations in cell-cell communication at the graft interface; (3) the formation and functional establishment of plasmodesmata at the graft interface; (4) the establishment of vascular reconnections between the scion and the rootstock (Yin et al., 2012; Melnyk, 2017; Melnyk et al., 2018; Rasool et al., 2020; Feng et al., 2024). Therefore, any abnormalities in molecular regulation or structural developmental defects at any stage of grafting may result in graft incompatibility.

It is due to the complexity of the mechanisms underlying graft incompatibility that its manifestations are highly diverse. Some forms of incompatibility can lead to the rapid wilting and death of the scion within a short timeframe, whereas others may initially appear compatible but eventually result in poor growth of the grafted plant over time. Specific symptoms include gradual swelling at the graft union, either in the scion or rootstock, reddish-brown discoloration of scion leaves, stunted growth and development of the scion, dwarfing, and increased susceptibility to breakage at the graft interface under external force (Loupit and Cookson, 2020). The timing of incompatibility onset also varies significantly. Certain cases can be detected within one to two days after grafting (DAG), while others remain undetectable in the short term, particularly in fruit tree grafting, where obvious incompatibility phenotypes may only become apparent after one year or even several years (Pina et al., 2017). Therefore, graft incompatibility has been broadly categorized into acute and delayed types. Under normal conditions, the diagnosis of graft compatibility or incompatibility is typically conducted through phenotypic observation after grafting (Tedesco et al., 2020; Xiong et al., 2021; Adhikari et al., 2022; Thomas et al., 2023) and chlorophyll fluorescence imaging in grafted melon (Calatayud et al., 2013). However, for delay incompatibility, this method is time-consuming and associated with relatively low accuracy.

Compared with compatible grafting, incompatible grafting induces a greater production of stress response compounds, such as reactive oxygen species (ROS) and hydrogen peroxide (H_2_O_2_) (Assunção et al., 2019). Therefore, as a result of the stress induced by incompatibility, secondary metabolites tend to accumulate substantially at the graft interface in cases of incompatible grafting (Errea, 1998). This insight has prompted researchers to explore the possibility of detecting and analyzing these secondary metabolites at the graft junction as a means to predict graft compatibility with relatively high accuracy and reliability prior to the grafting failure (Loupit et al., 2022). The initial report suggested that when pear and quince are grafted, cyanogenic glycosides present in the quince rootstock may transfer to the pear scion, where they are subsequently hydrolyzed into cyanide, ultimately resulting in graft failure five years after grafting (Gur et al., 1968; Moore, 1984). Subsequently, in the incompatible grafting of Eucalyptus gunnii, a significant accumulation of gallic acid, ellagic acid, gentisic acid, p-coumaric acid, and (+)-catechin was observed in pear/quince two years after grafting (De Cooman et al., 1996). Secondary metabolites accumulated in the graft interface of incompatible scion/rootstock combinations include 4-Hydroxyphenylacetic acid, Arbutin, Catechin, Ellagic acid, Epicatechin, Ferulic acid, Flavonoids dimers, Gallic acid, Gentisic acid, p-coumaric acid, Prunasin, Sinapic acid, etc., as summarized in the review by Loupit in 2020 (Loupit and Cookson, 2020). In addition, in 2022, stilbenes such as trans-resveratrol, trans-piceatannol, and α-viniferin were identified as potential metabolite markers for assessing grafting success. These compounds were measured one year after grafting in grapevines, specifically at the graft interface and surrounding woody tissues (Loupit et al., 2022; 2023). Meanwhile, the significant accumulation of primary metabolites, such as γ-aminobutyric acid (GABA), proline (Pro), and aspartic acid (Asp), along with secondary metabolites, including ferulic acid, naringenin, and taxifolin, at the graft interface may serve as potential markers for incompatibility (Loupit et al., 2022). Additional approaches have been explored for the early identification of graft incompatibility, such as phenol analysis, isozyme profiling, and chlorophyll fluorescence imaging (ur Rehman et al., 2025). Early detection of graft incompatibility enables the prompt screening of plants that are unlikely to survive after grafting, which is crucial for preserving important germplasm resources and breeding via grafting. To date, all these markers have been identified at the graft junction. Therefore, developing relatively precise diagnostic methods and efficient techniques for early identification of delayed graft incompatibility constitutes one of the critical challenges in the field of horticultural crop grafting.

While graft interface sampling has been the dominant strategy for identifying incompatibility markers, an emerging body of evidence suggests that the metabolic state of scion leaves can systematically reflect the physiological status of the graft junction. Following grafting, the rootstock and scion establish intimate vascular connections through which water, mineral nutrients, sugars, hormones, and signaling molecules are continuously exchanged. Recent studies have demonstrated that rootstock–scion interactions induce long-distance systemic effects, including the translocation of mobile mRNAs, small RNAs, and phloem sap metabolites, which significantly alter the transcriptome and metabolome of distal tissues. For instance, Davoudi et al. (2022) revealed that pumpkin rootstock exerts long-distance control over cucumber scion through the transmission of mobile mRNAs under drought stress. Furthermore, Tedesco et al. (2021) conducted comprehensive metabolomic profiling of eleven grapevine graft combinations and demonstrated that heterologous rootstocks significantly reshape the metabolic profiles of scion leaves, stems, and phloem exudates, indicating that the scion leaf metabolome serves as a sensitive recorder of below-ground grafting partner effects. Incompatible grafting induces local oxidative stress, phenolic accumulation, and cell death at the junction, which are associated with systemic disturbances in carbohydrate partitioning, hormone signaling, and secondary metabolism. These disturbances are ultimately transmitted to aerial organs, including scion leaves, where they manifest as measurable metabolic perturbations. Therefore, metabolites detected in scion leaves may not merely represent passive by-products of local leaf metabolism but rather serve as systemic biomarkers that integrate the rootstock–scion communication status. This conceptual framework provides a strong rationale for using scion leaves as a non-invasive, readily accessible material for the early prediction of delayed graft incompatibility.

The primary aim of this research is to use metabolomics approaches to identify metabolic markers in scion leaves that can enable the early detection of graft incompatibility. Unlike the graft interface, scion leaves are more readily obtainable and provide sufficient material for analysis. The identification marker metabolites in scion leaves for the early evaluation of delayed graft incompatibility not only helps mitigate economic losses and reduce production costs but also contributes to advancing the grafting industry.

## 2 Materials and methods

### 2.1 Plant growth, grafting and treatment

Tomato (cultivar Zhongshu No. 4) and pepper (cultivar Hangjiao No. 1) were selected as experimental materials. Seeds of tomato and pepper were soaked in sterile water at room temperature for 24 hours and sown in soil mixture (substrate soil: vermiculite: perlite = 10:1:1) after several times washing under controlled environmental conditions of 23-25 °C, a photoperiod of 16 h light/8 h dark, and relative humidity maintained at 60-70%. For TT self-grafting, 14-day-old tomato seedlings are selected. One day prior to grafting, the seedlings are watered thoroughly. During the grafting procedure, a double-sided blade is utilized to create an oblique cut at the internode located between the cotyledon and the first true leaf. Subsequently, a grafting clip is employed to secure the scion and rootstock together at the oblique cut site. A short plastic stake is then inserted adjacent to the grafted plant to provide structural support. Following the completion of the grafting process, a semi-transparent plastic dome is placed over the plant. The culture conditions in the growth chamber are maintained at 23-25°C, with a humidity level of 90%, and a photoperiod of 16h light/8h dark is sustained continuously.

For TP heterografting, 21-day-old pepper seedlings were used as rootstocks and 14-day-old tomato seedlings as scions, both of them with the same stem diameter. Oblique cuts were made at the same internodal position as described above, and the grafted plants were secured with grafting clips and covered with a semi-transparent plastic dome. The cultivation conditions were identical to those used for self-grafting. This single time point was selected based on preliminary observations indicating that metabolic perturbations precede visible incompatibility symptoms, which typically manifest after 7–10 DAG (Thomas et al., 2024). On the 5 DAG, the fourth true leaf of the tomato scion samples was collected from both TT and TP plants for subsequent analysis.

For treatment experiment, the cut end of the scion was immersed in the corresponding exogenous treatment solution for 30 seconds prior to grafting, after which grafting was performed according to the previously described method. The compounds utilized in our treatment experiment included the following: Trehalose (Analytical Pure, Shanghai Yuanye Biotechnology Co., Ltd.), Glucose and Sucrose (Analytical Pure, Xilong Science & Technology Co., Ltd.), Mannose (Analytical Pure, Shandong Baixing Biotechnology Co., Ltd.), Apigenin (Analytical Standard), Apigenin Glucoside (Analytical Standard), and Toxicodendron vernicifluum Glycoside (Analytical Standard). Notably, Apigenin, Apigenin Glucoside, and Toxicodendron vernicifluum Glycoside were procured from Chengdu Pufei Technology Development Co., Ltd. All compounds were prepared as stock solutions following the standard protocol and subsequently diluted to the required working concentrations. The selected concentration was based on preliminary dose-response experiments and concentrations previously reported in plant studies to elicit biological effects.

### 2.2 Bend test and leaf size analysis

To evaluate the grafting compatible, the bend test was employed to calculate the percentage of break (Thomas et al., 2022, 2023). Both of TT and TP were grafted thirty seedlings per replicate, with six replicates conducted in total, resulting in an overall sample of 180 grafting seedlings. Given that the number of biological replicates vary dependent on the variable nature of different graft survival, the TT group comprised 172 biological replicate samples, whereas the TP group included 63 samples in the bend test conducted on day 15, and the TT group consisted of 170 samples, while the TP group contained 16 samples in the bend test performed on day 30. To analyze the leaf size for both TT and TP, the fourth leaves of TT grafting (n = 74) and TP grafting (n = 74) were collected, and the length (L) and width (W) of the fourth true leaf of the scions were measured at the 5 DAG. Leaf length was defined as the distance from the base of the main vein to the leaf tip, while leaf width was defined as the length of the widest part of the leaf. All measurements were taken using a ruler.

### 2.3 Sample preparation and metabolomics analysis

Lyophilize leaf samples (TT and TP) using a lyophilizer (Scientz-100F). Grind the lyophilized leaves into powder at 30 Hz for 1.5 min using a grinder (MM 400, Retsch). Accurately weigh 50 mg of the sample powder on an electronic balance (MS105DM), and add 1200 μL of pre-cooled (-20 °C) 70% methanolic aqueous internal standard extract (at a ratio of 1200 μL extractant per 50 mg sample). Vortex the mixture for 30 sec every 30 min, repeating this process six times. Centrifuge the mixture at 12000 rpm for 3 min. Aspirate the supernatant, filter it through a 0.22 μm microporous membrane, and transfer the filtrate to an injection vial for ultra-performance liquid chromatography-tandem mass spectrometry (UPLC-MS/MS) analysis (Chen et al., 2013).

Sample extracts were analyzed using a UPLC-ESI-MS/MS system (UPLC: ExionLC™ AD, SCIEX; MS: Applied Biosystems 4500 Q TRAP, SCIEX). The analytical conditions were as follows: UPLC: Column, Agilent SB-C18 (1.8 µm, 2.1 mm × 100 mm); Mobile phase, Solvent A (pure water with 0.1% formic acid) and Solvent B (acetonitrile with 0.1% formic acid); Gradient program, starting with 95% A and 5% B, linearly adjusted to 5% A and 95% B within 9 min, maintained at 5% A and 95% B for 1 min, then returned to 95% A and 5% B within 1.1 min and held for 2.9 min; Flow rate, 0.35 mL/min; Column oven temperature, 40 °C; Injection volume, 4 µL. Mass Spectrometry: The effluent was directed to an ESI-triple quadrupole-linear ion trap (QTRAP)-MS for analysis.

The ESI source operation parameters were as follows: source temperature, 550 °C; ion spray voltage (IS), 5500 V (positive ion mode) or −4500 V (negative ion mode); ion source gas I (GSI), gas II (GSII), and curtain gas (CUR) were set to 50 psi, 60 psi, and 25 psi, respectively; collision-activated dissociation (CAD) was set to high. QQQ scans were acquired as multiple reaction monitoring (MRM) experiments with collision gas (nitrogen) set to medium. Declustering potential (DP) and collision energy (CE) for individual MRM transitions were optimized further. A specific set of MRM transitions was monitored during each period according to the metabolites eluted within that period.

### 2.4 Data processing, PCA, HCA and correlation coefficients

Metabolic data were analyzed using multivariate statistical analysis techniques. Metabolite annotation was performed using the database, MWDB (Wuhan Metware Biotechnology Co., Ltd., Wuhan, China). Metabolite identification integrates multiple parameters, including accurate mass, characteristic fragment ions of tandem mass spectrometry, isotope distribution of fragment ions, and retention time (RT) of the metabolites. Through the proprietary intelligent secondary spectrum matching algorithm developed by Metware Company, the secondary spectra and RT of metabolites in the samples were systematically compared and analyzed against the reference data stored in the database. The mass spectrometry tolerance was set to 2 ppm, while the secondary mass spectrometry tolerance was established at 5 ppm. For metabolites lacking authentic standards, identification was achieved by comparing their tandem mass spectra with those available in public databases or literature. In cases where no reference tandem mass spectra were available, structural characteristics were inferred based on expert knowledge and professional experience (Xiao et al., 2021; Zhu et al., 2018).

Unsupervised principal component analysis (PCA) and orthogonal partial least squares discriminant analysis (OPLS-DA) were performed using the prcomp function in R (www.r-project.org). The data were unit-variance scaled prior to unsupervised PCA. Hierarchical cluster analysis (HCA) results for samples and metabolites were visualized as heatmaps with dendrograms. Pearson correlation coefficients (PCC) between samples were calculated using the cor function in R and visualized as heatmaps without dendrograms. Both HCA and PCC analyses were conducted using the R package ComplexHeatmap. For HCA, normalized signal intensities of metabolites (unit-variance scaled) were visualized as a color spectrum.

### 2.5 Differential metabolites selected and metabolites pathways enrichment

The differential metabolites were identified based on a three-step screening strategy: (1) VIP values > 1 from OPLS-DA; (2) absolute Log2FC ≥ 1.0; and (3) Student’s t-test p-value< 0.05 with Benjamini-Hochberg false discovery rate (FDR) correction (q< 0.05). The Benjamini-Hochberg procedure was applied to control the FDR given the large metabolite panel (n = 1,493). VIP values were extracted from the OPLS-DA results, which also included score plots and permutation plots, and were generated using the R package Metabo Analyst R. The data underwent log transformation (log_2_) and mean centering prior to OPLS-DA analysis. To avoid overfitting, a permutation test (200 permutations) was conducted. Identified metabolites were annotated against the KEGG (Kyoto Encyclopedia of Genes and Genomes) Compound database (http://www.kegg.jp/kegg/compound/), and the annotated metabolites were subsequently mapped to the KEGG Pathway database (http://www.kegg.jp/kegg/pathway.html). Pathways with significantly regulated metabolites were then subjected to MSEA (metabolite set enrichment analysis), and their significance was determined based on the p-values calculated using the hypergeometric test.

### 2.6 Software and statistical analysis

Data analysis and visualization were performed using GraphPad 9 (GraphPad Software Inc., La Jolla, CA, USA) and the Metware Cloud, a free online platform for data analysis (https://cloud.metware.cn). The cluster heat maps were generated employing the ComplexHeatmap toolkit in R software. The Unit Variance Scaling (UV) method, a statistical technique, was applied to standardize the original dataset by utilizing the mean and standard deviation of the metabolomics data. This procedure transforms the data to adhere to a standard normal distribution, characterized by a mean of 0 and a standard deviation of 1. Standardization involves centering the data by subtracting the mean and subsequently scaling it by dividing by the variable’s standard deviation. The formula is as follows:


$$x^{\prime }=\frac{x-\mu }{\sigma }$$

Here, µ denotes the mean, while σ signifies the standard deviation. Statistical differences between TT and TP plants, or treatment and un-treatment, were calculated using Student’s T-Test. Significance was determined as p< 0.05.

## 3 Results

### 3.1 Basic analysis of metabolomic data of leaves in TP and TT scion

**Figure 1:**
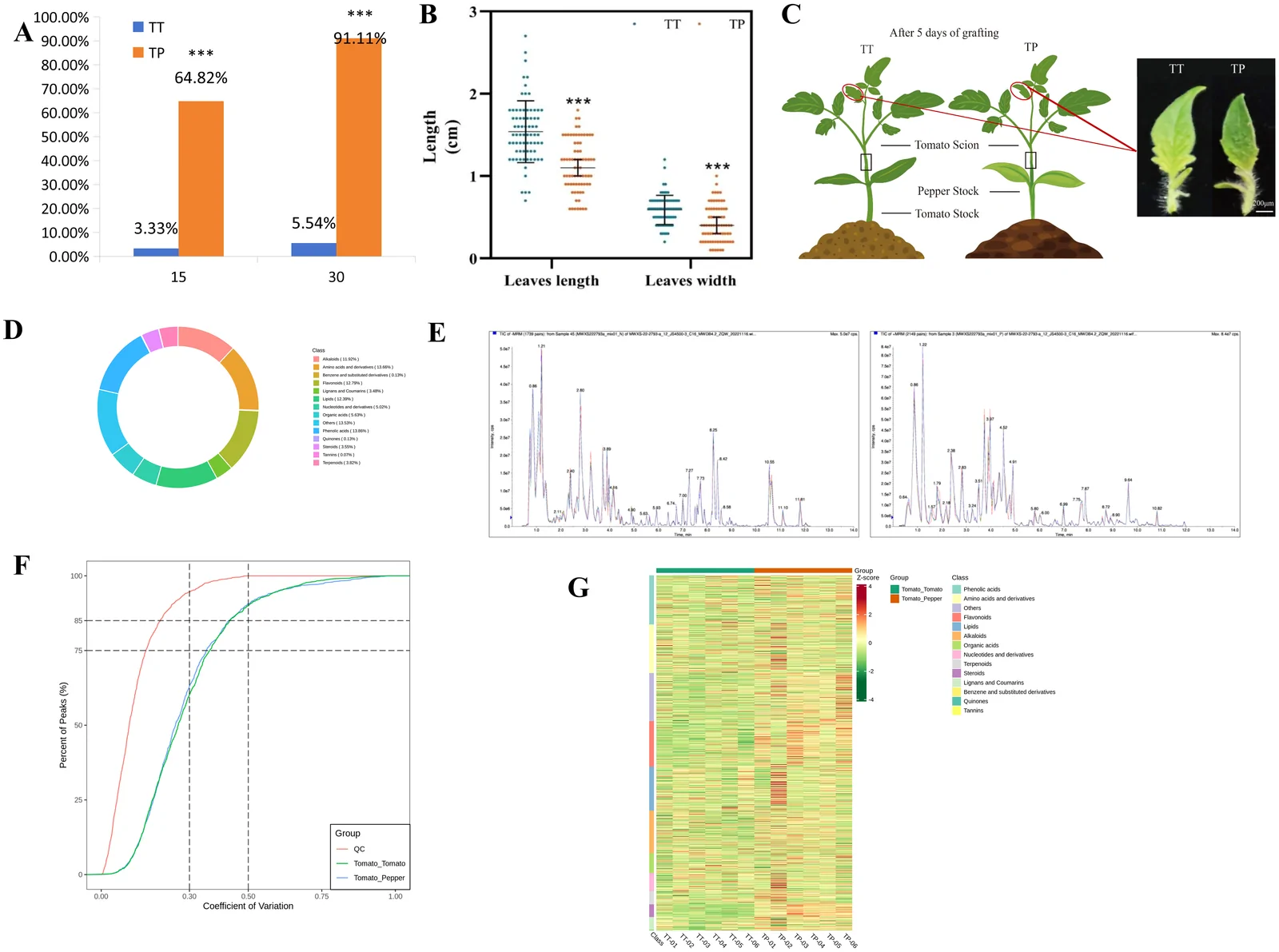
Sample preparation and Metabolites distribution in the Leaves of TT and TP Scions. **(A)** Percentage of Break of TT and TP at 15 and 30 days after grafting (DAG) by bend test. **(B)** Statistical analysis of the length and width of the fourth true leaf in TT and TP. Stars denote significant differences based on the Student’s t-test (p< 0.05). Three stars indicate P< 0.001. **(C)** Graphical depictions of the fourth true leaf sampling from TT and TP graft seedling. The red ellipse indicates the fourth true leaf. The inset on the right displays the fourth true leaf of TT and TP. The black rectangle indicates the graft junction site. **(D)** Circular diagram depicting the composition of metabolite categories. Each color corresponds to a specific metabolite category, and the size of the colored area reflects the proportion of that category within the dataset. **(E)** TIC Overlap for Mass Spectrometry Detection of QC Samples (N indicates negative ion mode; P indicates the positive ion mode). **(F)** Distribution of CV values for each group of samples. **(G)** Heatmap representing the clustering of overall samples. The horizontal axis represents the sample names, while the vertical axis indicates metabolite information. The term “Group” refers to the classification of samples. Cells are filled with different colors according to the standardized relative content values (z-score), with red representing high content and green representing low content. The term “Class” signifies the primary categorization of substances.

The grafting of tomato and pepper is a type of delay incompatibility grafting, with significant differences in compatibility among different variety combinations (Thomas et al., 2024). In this study, the tomato cultivar “Zhongshu No. 4” was utilized as the scion, while the pepper cultivar “Hangjiao No. 1” served as the rootstock. The results showed that the break rate percentage of the TP graft combination increased to 64.82% on the 15 DAG and further escalated to 91.11% by the 30 DAG (**Figure 1A**). Conversely, the break percentage of the TT remained consistently low at approximately 3.33-5.54% (**Figure 1A**). Given that the number of biological replicates vary dependent on the variable nature of different graft survival, the TT group comprised 172 biological replicate samples, whereas the TP group included 63 samples in the bend test conducted on 15 DAG, and the TT group consisted of 170 samples, while the TP group contained 16 samples in the bend test performed on 30 DAG. This progressive reduction in TP sample size is a direct consequence of the delayed incompatibility phenotype: tomato scions grafted onto pepper rootstocks exhibit progressively deteriorating graft union integrity, with the majority of grafted seedlings failing between 15 and 30 DAG. The precipitous decline in available TP samples at 30 DAG reflects the natural trajectory of incompatible graft failure rather than an experimental design limitation, thereby providing a phenotypic validation of the TP combination’s incompatibility. Within the first week after grafting, distinct anatomical differences emerged grafting junction in TP due to the delayed wound response of pepper rootstock compared with TT (Thomas et al., 2021). This result indicates that further investigation is warranted regarding whether the response at the junction of incompatible grafting influences the development and growth of scion leaves. Therefore, on the 5 DAG, the size of the fourth true leaves of TT and TP grafts examined in our research. Although no obvious phenotypic differences, such as plant height and stem diameter, were observed between TT and TP on the 5 DAG, there was a significant reduction in both the length and width of the fourth true leaves (**Figure 1B**). This suggests that a more comprehensive understanding of the changes in metabolome within the scion leaves may serve as a valuable indicator for evaluating delayed graft incompatibility.

To investigate the metabolic changes in the scion leaves during the early stages of delay graft incompatibility, we collected the fourth leaf of the tomato scion on the 5 DAG as the subject of our study (see **Figure 1C**). In the experimental design, the fourth leaf of the scion from the TT compatible graft combination harvested at the 5 DAG served as the control group, whereas the fourth leaf of the scion from the TP delay incompatible graft combination harvested at the 5 DAG was designated as the experimental group. Each group consisted of six biological replicates, totaling 12 samples for subsequent analysis (see **Table 1**). All samples were processed according to the procedural steps outlined in the methods section and analyzed using UPLC-MS/MS. Initially, the total ion current (TIC) chromatogram of the mixed quality control (QC) samples was generated (**Supplementary Figure 1**), and the detectable substances within the samples were represented as MRM metabolite detection multi-peak chromatograms, specifically the extracted ion chromatograms (XICs) of multiple compounds (**Supplementary Figure 2**). To compare the content differences of each metabolite in TT and TP leaves, the chromatographic peaks of detected metabolites across different samples were normalized based on retention time and peak shape characteristics, ensuring the accuracy of both qualitative and quantitative analyses. The results of random sampling for metabolite quantification and integration correction across different samples are presented in **Supplementary Figure 3**. Comprehensive details regarding all detected metabolites are summarized in **Supplementary Table 1**. The findings revealed that a total of 1,493 metabolites were identified, with their composition being sample-specific and exhibiting variations in both types and proportions among different samples. Through compositional proportion analysis, the overall distribution of major metabolites within the samples was clarified (**Figure 1D**). The results showed that the detected metabolites included phenolic acids (13.86%), amino acids and derivatives (13.66%), lipids (12.39%), flavonoids (12.79%), alkaloids (11.92%), organic acids (5.63%), nucleotides and derivatives (5.02%), terpenoids (3.82%), steroids (3.55%), lignans and coumarins (3.48%), as well as benzene and its substituted derivatives (0.13%), quinones (0.13%), and tannins (0.07%). Additionally, sugars were classified under other class (13.53%), accounting for 55.45% (112/202) of this category.

**Table 1:** Samples and groups information.

Group (scion/rootstocks)	Samples ID	Tissues	Species
tomato/tomato	TT-01	leaves	tomato
	TT-02	leaves	tomato
	TT-03	leaves	tomato
	TT-04	leaves	tomato
	TT-05	leaves	tomato
	TT-06	leaves	tomato
tomato/pepper	TP-01	leaves	tomato
	TP-02	leaves	tomato
	TP-03	leaves	tomato
	TP-04	leaves	tomato
	TP-05	leaves	tomato
	TP-06	leaves	tomato

QC samples are prepared by blending sample extracts and are used to evaluate the repeatability of sample analysis under identical processing conditions. During instrumental analysis, a QC sample is inserted after every 10 samples to continuously monitor the stability and reproducibility of the analytical process in real time. By overlaying the TIC of different QC samples (**Figure 1E**; **Supplementary Figure 4**), it is evident that the metabolite detection TIC exhibit a high degree of consistency, confirming stable retention times and peak intensities. This indicates that the MS maintains excellent signal stability when analyzing the same sample at different times. The coefficient of variation (CV value), defined as the ratio of the standard deviation to the mean of the original data, serves as an effective metric for quantifying data dispersion. Using the empirical cumulative distribution function (ECDF), the frequency of CV values below the reference threshold can be further analyzed (**Figure 1F**; **Supplementary Figure 5**). A higher proportion of lower CV values reflects enhanced experimental data stability. Specifically, when more than 85% of substances in the QC samples have CV values less than 0.5, it suggests relatively stable experimental data; the results of this study demonstrate that over 75% of substances in the QC samples have CV values less than 0.3, indicating exceptionally high stability of our experimental data.

To elucidate the overall metabolite differences among the TT and TP groups and the variability within them, PCA was performed on all samples, including QC samples (**Supplementary Figure 4**; **Supplementary Tables 2**, **3**). This analysis provided the explanation rates of the top five principal components for the entire dataset and generated the PC1 control chart for all samples (**Supplementary Figures 5**, **6**). Subsequently, the data were subjected to UV normalization, and a clustering heatmap was constructed for all samples (**Figure 1G**). Thereafter, inter-sample correlation analysis was conducted to evaluate the biological reproducibility of samples within each group (**Supplementary Figure 7**; **Supplementary Table 4**). The results demonstrated that the correlation coefficients within each group were significantly higher than those between groups, thereby confirming the high reliability and quality of the identified differential metabolites.

### 3.2 Differential analysis of metabolites in the leaves of TP-incompatible scions

**Figure 2:**
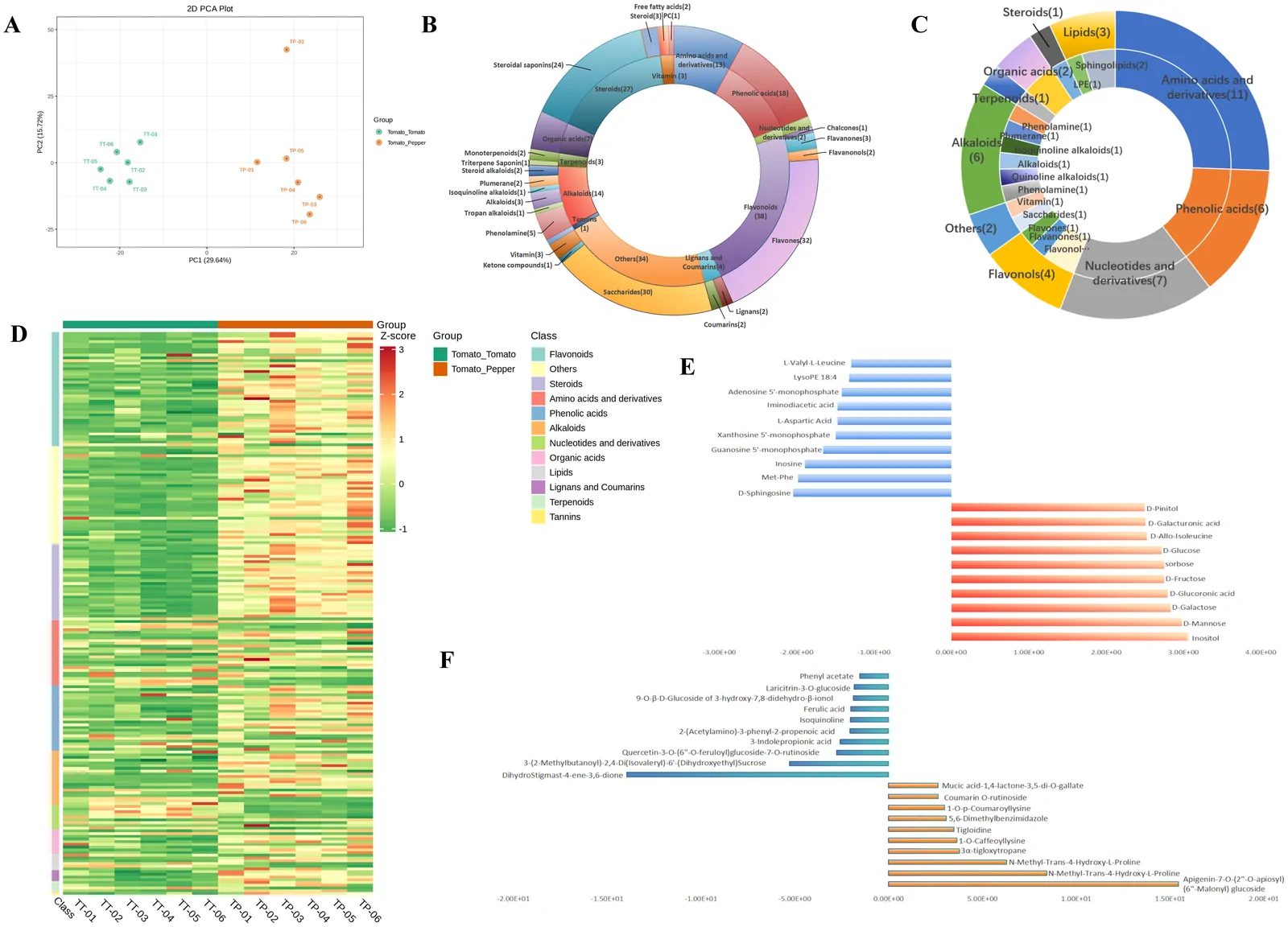
Analysis of differential metabolites in leaves of TP scion. **(A)** PCA plot illustrating sample grouping within TT and TP group. PC1 represents the first principal component, PC2 represents the second principal component, and the percentages reflect the proportion of variance explained by each principal component in the dataset. Each point corresponds to an individual sample, with samples from the same group indicated by a consistent color. The grouping information is labeled as “Group.” **(B, C)** Circular diagram depicting the composition of differential metabolite class accumulated in TP **(B)** and reduced in TP **(C)**. Each color corresponds to a specific class of metabolites, and the size of the colored area reflects the proportion of that class within the dataset. **(D)** Heatmap representing the clustering of differential metabolites in leaves of TT and TP. The horizontal axis denotes the sample names, while the vertical axis indicates metabolite information. The term “Group” refers to the classification of samples. Cells are filled with different colors according to the standardized relative content values (z-score), with red representing high content and green representing low content. The term “Class” signifies the primary categorization of substances. **(E, F)** Bar chart representing top 10 primary **(E)** and secondary **(F)** metabolites. The x-axis represents the log_2_ fold change (FC) of differential metabolites, which is the base-2 logarithmic value of the fold change in metabolite levels. The y-axis lists the differential metabolites. Bars extending to the right represent an increase in metabolite content, whereas bars extending to the left bar indicates decreased content.

To investigate the differential metabolites and evaluate the extent of variation both within and among the TT and TP groups, PCA (**Figure 2A**; **Supplementary Figures 8**, **9**; **Supplementary Tables 5**, **6**) and OPLS-DA (**Supplementary Figures 10**, **11**) were conducted on the all samples of TT and TP. The PCA of entire data set results indicated that the contribution rates of PC1 and PC2 to the samples were 29.64% and 15.72% (**Figure 2A**), respectively, reflecting the primary characteristic information of the samples. The clear separation of the samples along the horizontal coordinate axis demonstrated the reliability of the data processing outcomes and their ability to represent the metabolic differences between the TT and TP groups. The OPLS-DA results revealed that the contribution rates of the first and second principal components were 29.9% and 10.2%, respectively (**Supplementary Figure 10A**). The data from the two groups were distinctly clustered on either side of the confidence interval, confirming a robust discrimination effect and reliable data. In the OPLS-DA validation model, Q^2^ serves as an indicator of the model’s predictive capacity, with values greater than 0.9 signifying an excellent model. In this study, R^2^X was 0.401, R^2^Y was 0.998, and Q^2^ was 0.916 (**Supplementary Figure 10B**), indicating that the model exhibited exceptional predictive ability and reliability, effectively capturing the metabolic differences between the TT and TP groups. Meanwhile, an OPLS-DA model with no fewer than six biological replicates was employed to calculate the variable importance in projection (VIP) for the preliminary identification of differential metabolites between the TT and TP group (**Supplementary Figure 11**). Furthermore, fold change (FC) values were calculated to provide additional validation of these differential metabolites. The detailed screening criteria for identifying differential metabolites in this study are outlined in the Methods section.

Based on the aforementioned screening criteria, our study identified a total of 207 differential metabolites, including 164 up-regulated and 43 down-regulated metabolites (**Table 2**; **Supplementary Table 7**).

**Table 2:** The number of differential metabolites in the leaves of Tomato_Tomato vs Tomato_Peppe.

Group name	Total	Reduced in TP	Accumulated in TP
TT_vs_TP	207	43	164

TT_vs_TP refers to the differential metabolites identified in TP when compared to TT, which serves as the control group.

The 207 differential metabolites were initially screened using the standard pipeline (VIP > 1 and |Log_2_FC| ≥ 1) provided by Metware Biotechnology. To further evaluate statistical rigor, we independently performed univariate t-tests with Benjamini–Hochberg FDR correction. Notably, 148 out of 207 metabolites (71.5%) remained significant at FDR< 0.05, confirming that the majority of our identified markers are statistically robust. The full FDR-corrected results are provided in **Supplementary Table 8** (**Supplementary Table 8**).

Classification analysis of these differential metabolites (**Figures 2B, C**) revealed significant differences across various categories: flavonoids (n = 42, with 38 accumulated and 4 decreased), sugars (others class, n = 31, with 30 accumulated and 1 decreased), steroids (n = 28, with 27 accumulated and 1 decreased), amino acids and their derivatives (n = 24, with 13 accumulated and 11 decreased), phenolic acids (n = 24, with 18 accumulated and 6 decreased), alkaloids (n = 20, with 14 accumulated and 6 decreased), nucleotides and their derivatives (n = 9, with 2 accumulated and 7 decreased), organic acids (n = 9, with 7 accumulated and 2 decreased), lipids (n = 6, with 3 accumulated and 3 decreased), vitamins (n = 4, with 3 accumulated and 1 decreased), terpenoids (n = 4, with 3 accumulated and 1 decreased), and lignans and coumarins (n = 4, all accumulated). Especially, lignans and coumarins, tannins, and ketone compounds were exclusively accumulated in the TP group. Among the accumulated metabolites, flavonoids constituted the largest category (n = 38), with flavonols accounting for the highest proportion (n = 21); among the reduced metabolites, amino acids and their derivatives were the most numerous (n = 11) (**Figures 2B, C**). To visually represent the variation patterns of the relative contents of differential metabolites, this study employed the original relative content data of differential metabolites selected by UV processing (for the specific calculation formula, see Methods) to construct a heatmap using the R software package (**Figure 2D**). Among all accumulated compounds, Apigenin-7-O-(2’’-O-apiosyl) (6’’-Malonyl) glucoside exhibited the largest fold change in the TP group; among the decreased compounds, DihydroStigmast-4-ene-3,6-dione demonstrated the largest fold change in the TP group (**Supplementary Table 7**).

To comprehensively analyze the characteristics of differential metabolites in the leaves of scions from incompatible grafts, this study conducted a systematic investigation into primary and secondary metabolites (**Supplementary Tables 9**, **S10**). A total of 68 significantly differential primary metabolites were identified, including of 46 accumulated and 22 decreased compounds. Based on log2FC values, the top 10 accumulated and decreased metabolites with the highest fold changes were selected (**Figure 2E**). Among the top 10 accumulated metabolites, nine were sugars and one was an amino acid derivative. The top 10 decreased metabolites included 3 amino acids and their derivatives, 4 nucleotides and their derivatives, 2 lipids, and 1 organic acid. A total of 139 significantly differential secondary metabolites were detected in the TP group, comprising 118 accumulated and 21 decreased compounds. Similarly, based on log2FC values, the top 10 accumulated and decreased metabolites with the highest fold changes were selected (**Figure 2F**). The top 10 accumulated metabolites included 1 flavonoid, 1 sugar, 1 amino acid derivative, 5 alkaloids, 1 coumarin, and 1 phenolic acid. The top 10 decreased metabolites included 1 steroid, 1 carbohydrate, 2 flavonoids, 3 alkaloids, 2 phenolic acids and 1 terpene.

### 3.3 Analysis of KEGG metabolic pathways for differential metabolites in the leaves of TP

**Figure 3:**
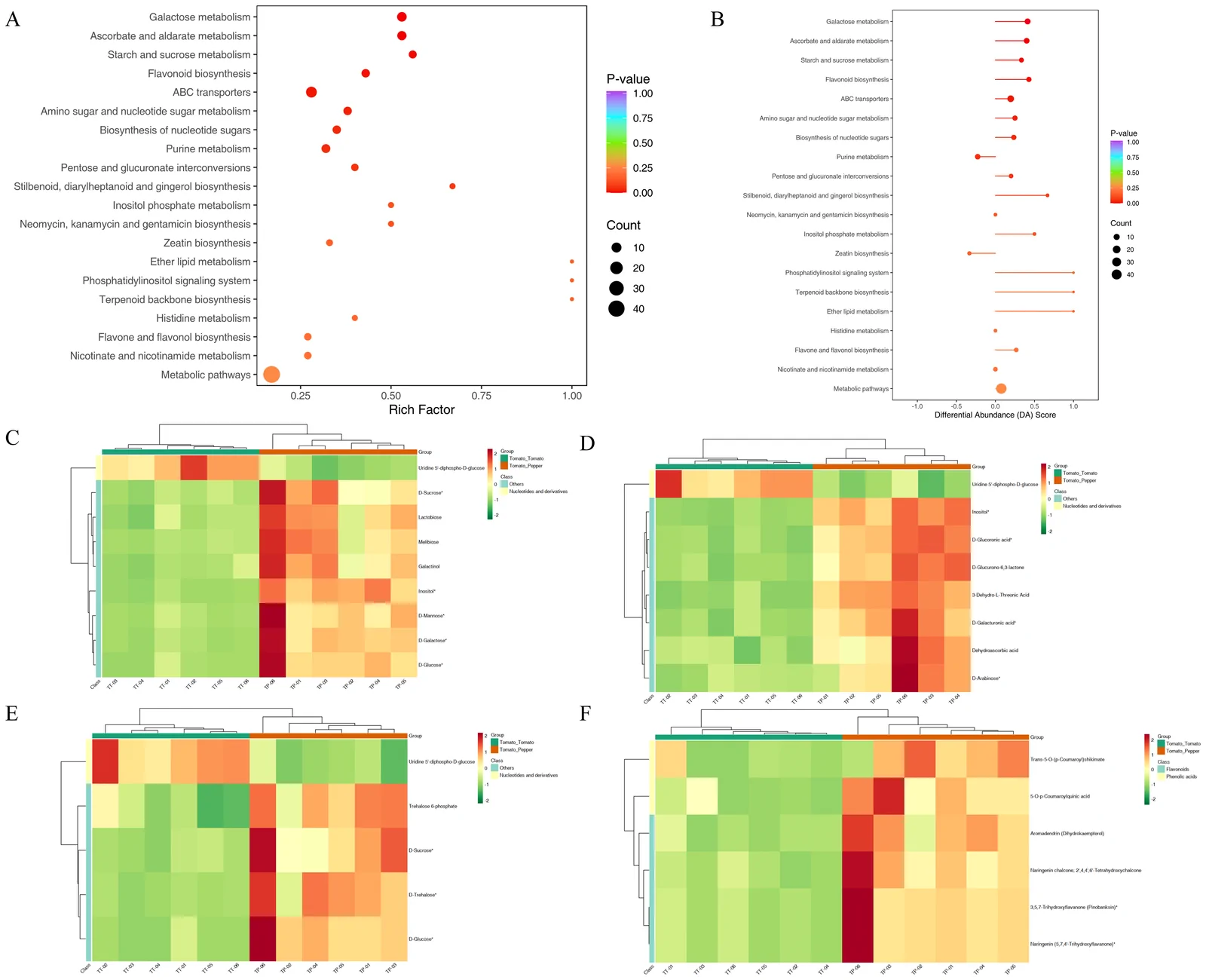
KEGG Metabolic Pathway Enrichment Analysis of Differential Metabolites in TP Leaves. **(A)** Top 20 metabolic pathway enrichment. The horizontal axis represents the rich factor associated with each pathway, while the vertical axis lists the pathway names (sorted by P-value). The color intensity of the dots reflects the magnitude of the P-value, with deeper red hues representing more significant enrichment. The dots size corresponds to the number of differentially expressed metabolites enriched in each pathway. **(B)** Plot of differential metabolite abundance scores (DA score). The vertical axis displays the names of the differential pathways, sorted by their P-values, while the horizontal axis indicates the DA Score. The DA Score quantifies the overall change in all metabolites within a metabolic pathway. A score of 1 signifies that all identified metabolites in the pathway were accumulated, whereas a score of -1 indicates reduction. The length of the line segment corresponds to the absolute value of the DA Score. The size of the dot at the end of the line segment reflects the number of differential metabolites in the pathway. If the dot is located on the left side of the central axis and the line segment is longer, it suggests that the overall pathway tends to be reduced. Conversely, if the dot is on the right side of the central axis and the line segment is longer, it implies that the pathway’s overall tends to be accumulated. Larger dots indicate a greater number of metabolites. The color of the line segment and the dot represents the magnitude of the P-value, with colors closer to red indicating smaller P-values and colors closer to purple indicating larger P-values. **(C–F)** Heatmap representation of differentially compounds clustering within galactose metabolism(ko00052) **(C)**, ascorbate and aldarate metabolism (ko00053) **(D)**, starch and sucrose metabolism (ko00500) **(E)** and flavonoid Biosynthesis (ko00941) **(F)**. The horizontal axis represents the sample names, while the vertical axis lists the differential metabolites. Cells are filled with varying colors based on standardized relative content values (red signifies high content, and green signifies low content). The annotation bar above the heatmap corresponds to the samples of TT and TP, with distinct colors representing TT and TP group (Group), and the dendrogram illustrates the clustering results of the differential samples. The annotation bar on the left side of the heatmap corresponds to the compounds, with distinct colors representing different substance class (Class), and the dendrogram illustrates the clustering results of the differential compounds.

**Figure 4:**
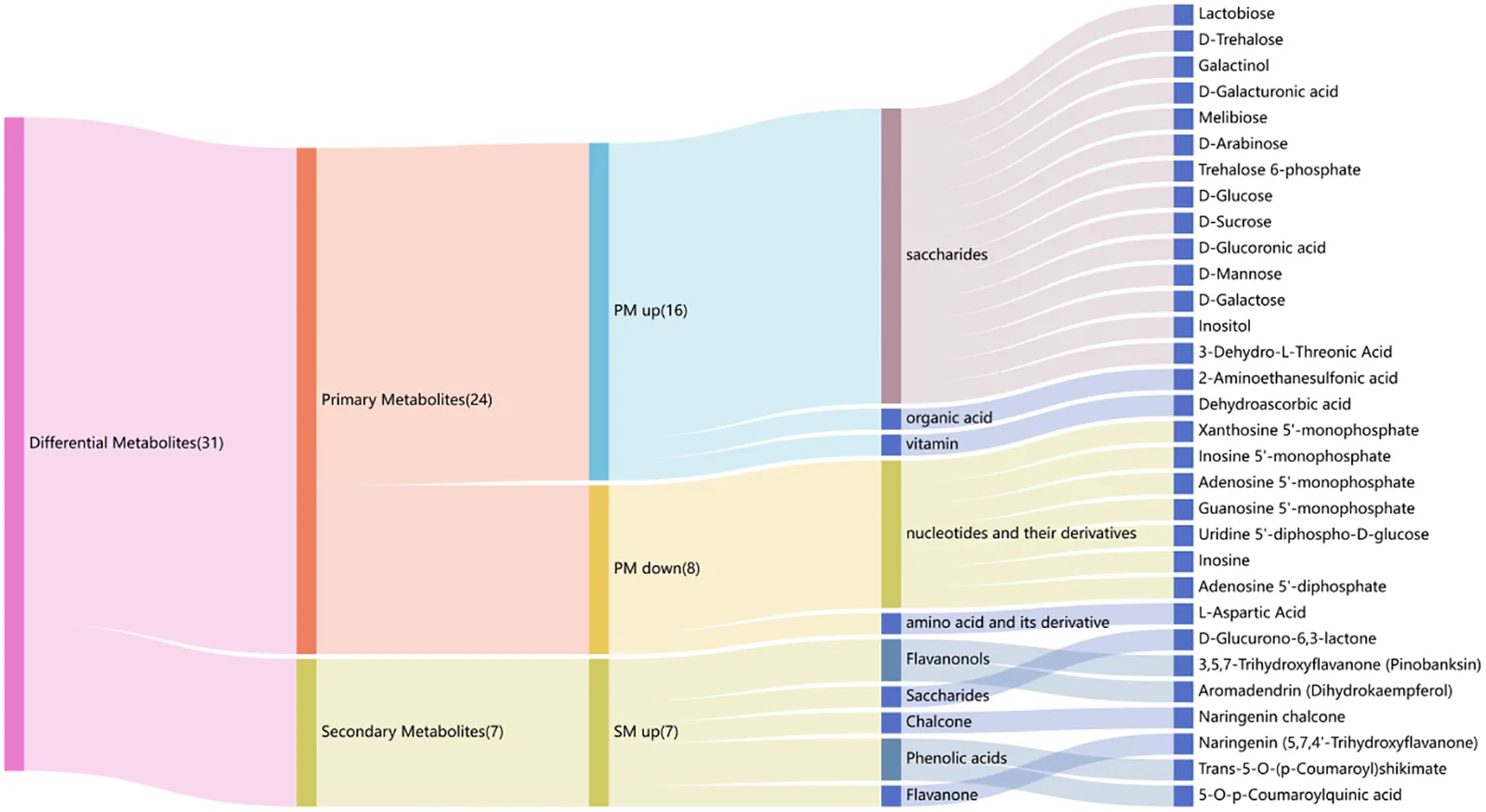
Sankey diagram visualizing the classification, accumulation and reduction of 31 differential metabolites. The width of the connections between each vertical block represents the number of metabolites in each class (from left to right). The numbers in parentheses indicate the counts of the respective metabolites. PM, primary metabolite; SM, secondary metabolite; up, accumulated metabolite; down, reduced metabolite. The names of 31 differential metabolites are listed on the far right of the image (from top to bottom).

**Figure 5:**
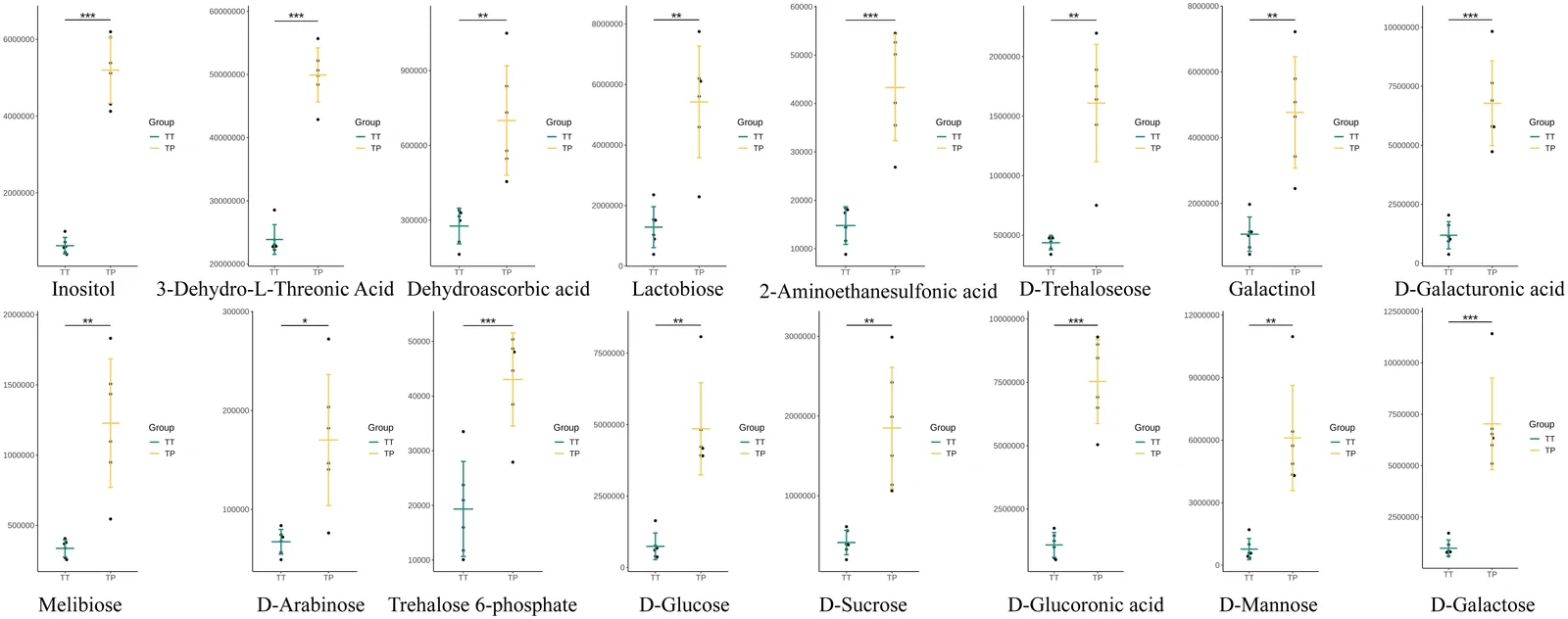
Beeswarm plot of 16 accumulated primary metabolites. The horizontal axis represents the groups, while the vertical axis reflects the relative abundance of differential metabolites.

**Figure 6:**
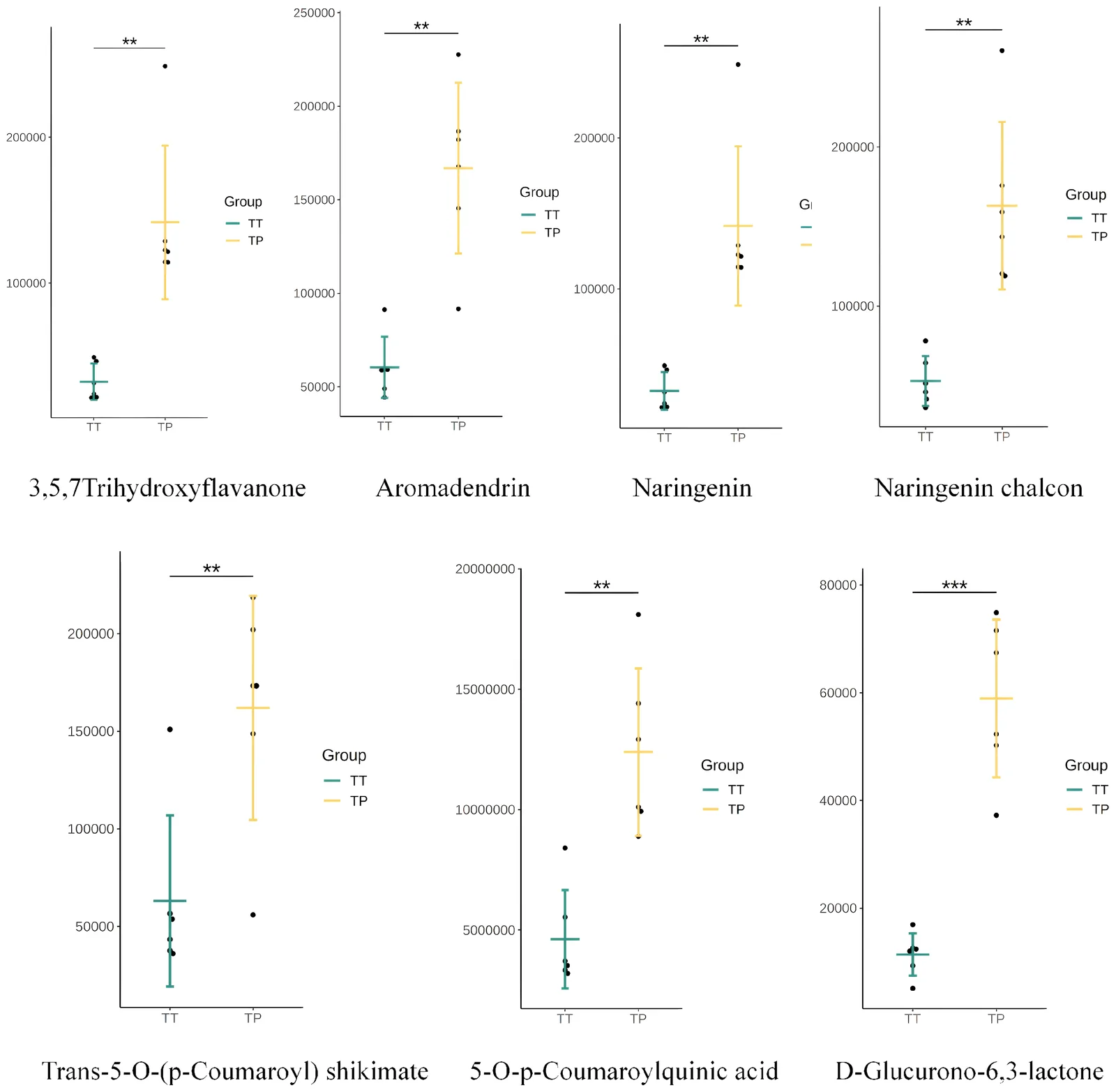
Beeswarm plot of 7 acumulated secondary metabolites. The horizontal axis represents the groups, while the vertical axis reflects the relative abundance of differential metabolites.

**Figure 7:**
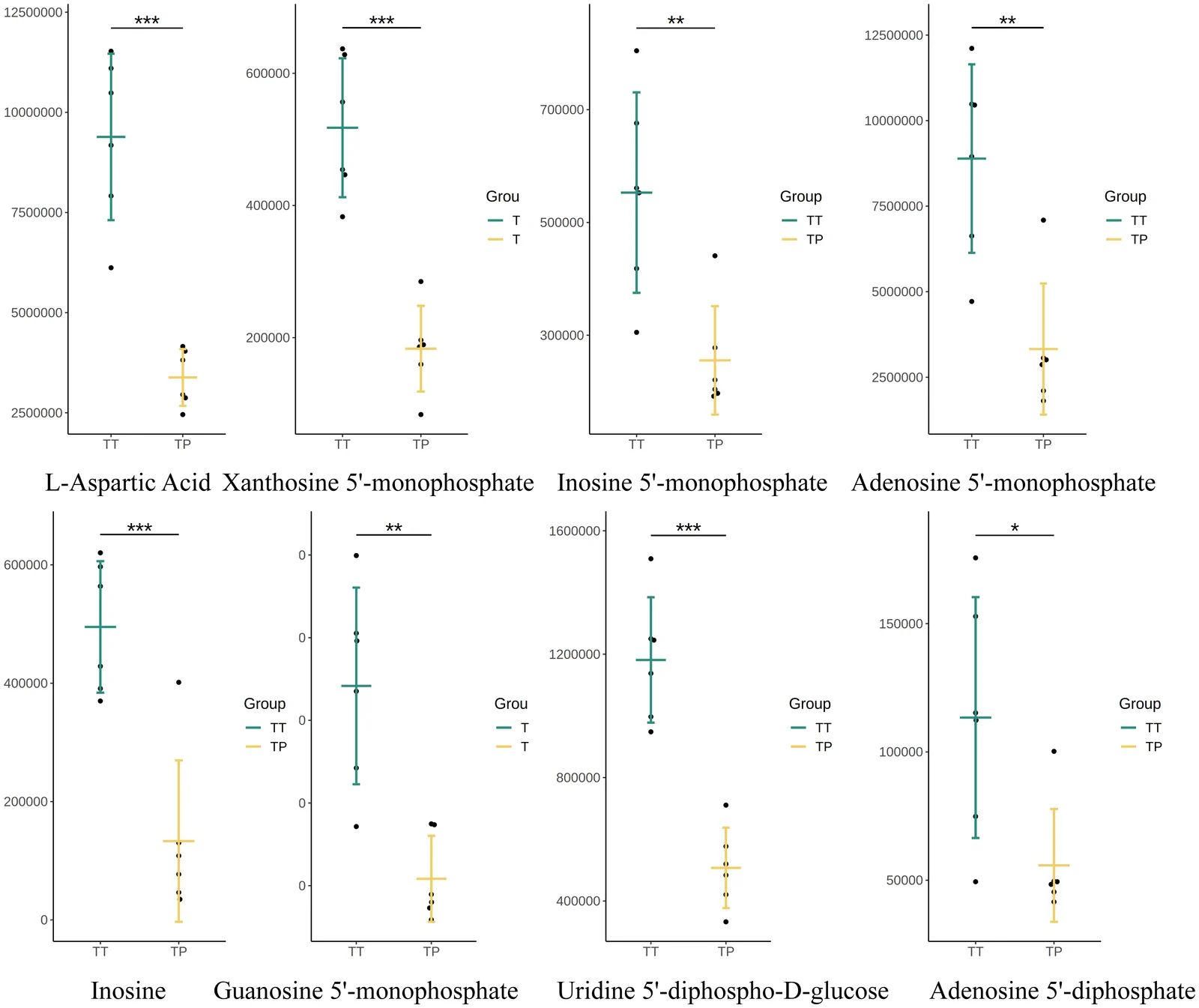
Beeswarm plot of 8 reduced primary metabolites. The horizontal axis represents the groups, while the vertical axis reflects the relative abundance of differential metabolites.

**Figure 8:**
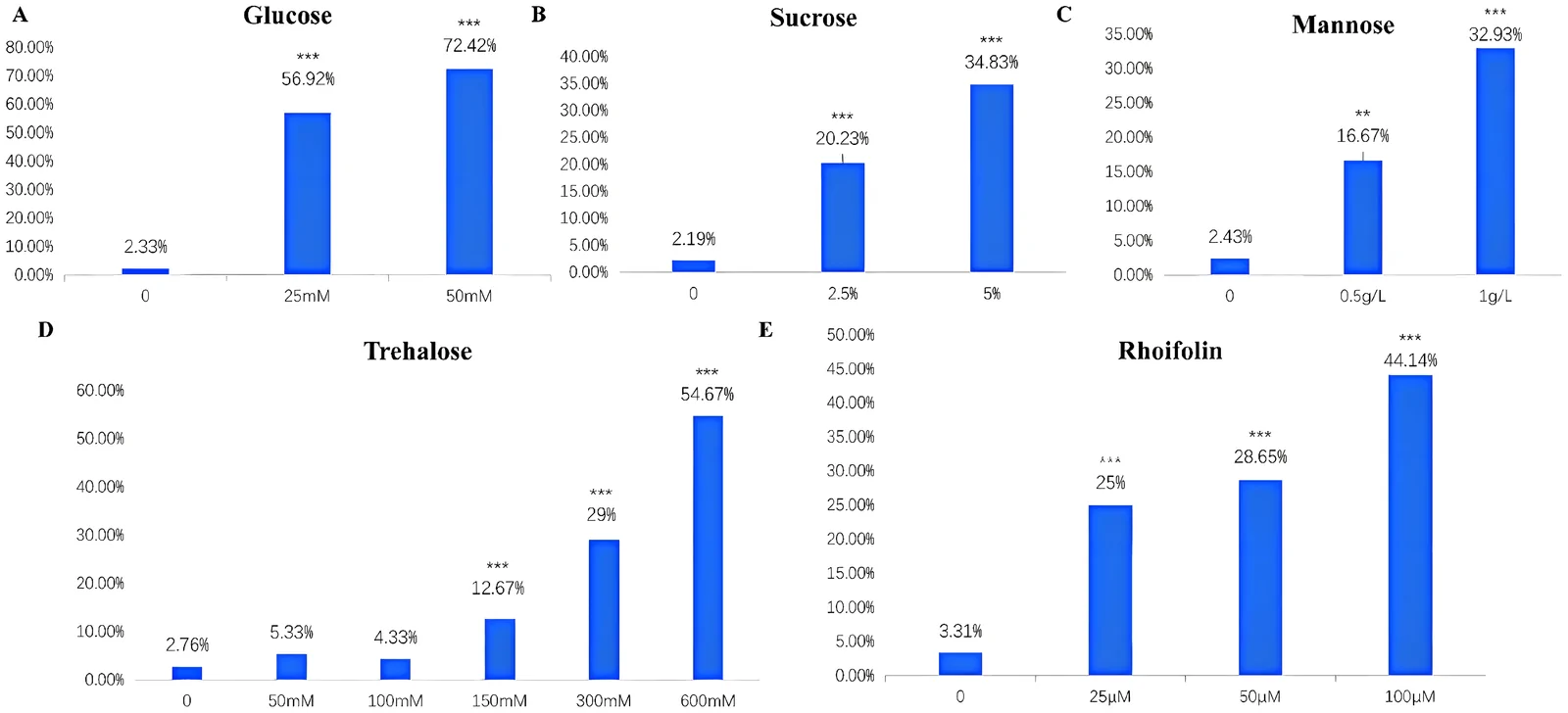
The Effect of Metabolites on the Break percentage of TT Compatible Grafting. **(A)** Glucose, **(B)** Sucrose, **(C)** Mannose, **(D)** Trehalose, **(E)** Rhoifolin. The x-axis denotes the concentration of metabolic compounds, the y-axis indicates the break percentage by bend test (%). Two asterisks indicate the significant differences according to the student t test at P< 0.01. Three asterisks indicate P< 0.001. Thirty seedlings were grafted per replicate, with six replicates performed in total, resulting in an overall sample size of 180 grafting seedlings used for treatment with each concentration of each compound.

To further elucidate the metabolic pathways associated with the differential metabolites in TP, KEGG metabolic pathway annotation was performed. The results revealed that 207 differential metabolites were mapped to 65 pathways (**Supplementary Figure 12**; **Table 2**). The top 20 enriched metabolic pathways, ranked by P-value, are presented in **Figure 3A**. To comprehensively understand the overall changes of all metabolites across metabolic pathways, the differential abundance score (DA Score) was calculated and visualized in **Figure 3B**. Eight KEGG pathways were ultimately identified as significantly enriched among all analyzed pathways (p< 0.05). The P-value, derived from the hypergeometric test, reflects the statistical significance of enrichment, where a smaller P-value indicates a higher level of enrichment significance. A total of 34 differential metabolites were enriched in these pathways (**Table 3**). To investigate the variation patterns of substance contents between TT and TP within the eight significantly enriched pathways, cluster analysis was performed on all differential metabolites identified in these enriched KEGG metabolic pathways (**Figures 3C–F**; **Supplementary Figure 13**). The results showed that, among the 34 metabolites identified, three were found to exhibit p-values greater than 0.05 via t-test statistical analysis in between TP and TT groups. Finally, a total of 31 metabolites (23 accumulated and 8 decreased in TP) may serve as characteristic metabolites associated with graft incompatibility (**Table 4**). Specifically, 24 primary metabolites (16 accumulated and 8 decreased in TP) and 7 secondary metabolites (all of them accumulated in TP) included (**Figures 4**–**7**). The 16 primary metabolites accumulated (**Figure 5**; **Tables 3**, **4**) including 14 saccharides, 1 organic acid, and 1 vitamin while the eight reduced (**Figure 8**; **Tables 3**, **4**) including seven nucleotides and their derivatives, as well as one amino acid and its derivative.

**Table 3:** Eight KEGG pathway significantly enriched in TT and TP grafting.

Metabolism	ko ID	KEGG pathway	P-value	Sig	Up compound	Down compound
Carbohydrate metabolism	ko00052	Galactose metabolism	0	9	D-Mannose; Galactinol; Melibiose; D-Galactose; Lactobiose; D-Glucose; D-Sucrose; Inositol	Uridine 5’-diphospho -D-glucose
	ko00053	Ascorbate and aldarate metabolism	0.001	8	D-Glucoronic acid; D-Arabinose; 3-Dehydro-L-Threonic Acid; D-Glucurono-6,3-lactone; D-Galacturonic acid; Inositol; Dehydroascorbic acid	Uridine 5’-diphospho -D-glucose
	ko00500	Starch and sucrose metabolism	0.006	5	Trehalose 6-phosphate;D-Glucose; D-Sucrose;D-Trehalose;	Uridine 5’-diphospho -D-glucose
Biosynthesis of other secondary metabolites	ko00941	Flavonoid biosynthesis	0.013	6	5-O-p-Coumaroylquinic acid; Trans-5-O-(p-Coumaroyl) shikimate; Naringenin chalcone 2’,4,4’,6’-Tetrahydroxychalcone; 3,5,7-Trihydroxyflavanone (Pinobanksin); Aromadendrin (Dihydrokaempferol); Naringenin (5,7,4’-Trihydroxyflavanone)	/
ABC transporters	ko02010	ABC transporters	0.015	13	2-Aminoethanesulfonic acid; L-Histidine; L-Proline; Melibiose; D-Mannose; Lactobiose; Inositol;D-Trehalose; D-Galacturonic acid; D-Glucose; D-Sucrose	Inosine; L-Aspartic Acid
Glycan biosynthesis and metabolism	ko00520	Amino sugar and nucleotide sugar metabolism	0.026	6	D-Glucoronic acid; D-Arabinose; D-Mannose; D-Galacturonic acid; D-Glucose	Uridine5’-diphospho-D-glucose
Global and overview maps	ko01250	Biosynthesis of nucleotide sugars	0.035	6	D-Glucoronic acid; D-Arabinose; D-Mannose;D-Galacturonic acid;Inositol	Uridine 5’-diphospho -D-glucose
Nucleotide metabolism	ko00230	Purine metabolism	0.041	7	Guanosine 3’,5’-cyclic monophosphate	Adenosine 5’- monophosphate; Adenosine 5’- diphosphate; Inosine; Inosine 5’- monophosphate; Guanosine 5’- monophosphate; Xanthosine 5’- monophosphate;

**Table 4:** The classification of 31 differential metabolites isolated from the leaves of TP scions.

Compounds	Formula	Class I	Class II	Type
L-Aspartic Acid	C_4_H_7_NO_4_	Amino acids and derivatives	Amino acids and derivatives	down
3,5,7-Trihydroxyflavanone	C_15_H_12_O_5_	Flavonoids	Flavanonols	up
(Pinobanksin)	C_15_H_12_O_6_	Flavonoids	Flavanonols	up
Aromadendrin (Dihydrokaempferol)	C_15_H_12_O_5_	Flavonoids	Flavanones	up
Naringenin (5,7,4’-Trihydroxyflavanone)	C_15_H_12_O_5_	Flavonoids	Chalcones	up
Naringenin chalcone; 2’,4,4’,6’-Tetrahydroxychalcone	C_10_H_13_N_4_O_9_P	Nucleotides and derivatives	Nucleotides and derivatives	down
Xanthosine 5’-monophosphate	C_10_H_13_N_4_O_8_P	Nucleotides and derivatives	Nucleotides and derivatives	down
Inosine 5’-monophosphate	C_10_H_14_N_5_O_7_P	Nucleotides and derivatives	Nucleotides and derivatives	down
Adenosine 5’-monophosphate	C_10_H_14_N_5_O_8_P	Nucleotides and derivatives	Nucleotides and derivatives	down
Guanosine 5’-monophosphate	C_15_H_24_N_2_O_17_P_2_	Nucleotides and derivatives	Nucleotides and derivatives	down
Uridine 5’-diphospho-D-glucose	C_10_H_12_N_4_O_5_	Nucleotides and derivatives	Nucleotides and derivatives	down
Inosine	C_10_H_15_N_5_O_10_P_2_	Nucleotides and derivatives	Nucleotides and derivatives	down
Adenosine 5’-diphosphate	C_2_H_7_NO_3_S	Organic acids	Organic acids	up
2-Aminoethanesulfonic acid	C_6_H_12_O_6_	Others	Saccharides	up
Inositol	C_4_H_6_O_5_	Others	Saccharides	up
3-Dehydro-L-Threonic Acid	C_6_H_6_O_6_	Others	Vitamin	up
Dehydroascorbic acid	C_12_H_22_O_11_	Others	Saccharides	up
Lactobiose	C_12_H_22_O_11_	Others	Saccharides	up
D-Trehalose	C_12_H_22_O_11_	Others	Saccharides	up
Galactinol	C_6_H_10_O_7_	Others	Saccharides	up
D-Galacturonic acid	C_12_H_22_O_11_	Others	Saccharides	up
Melibiose	C_5_H_10_O_5_	Others	Saccharides	up
D-Arabinose	C_12_H_23_O_14_P	Others	Saccharides	up
Trehalose 6-phosphate	C_6_H_12_O_6_	Others	Saccharides	up
D-Glucose	C_6_H_8_O_6_	Others	Saccharides	up
D-Glucurono-6,3-lactone	C_12_H_22_O_11_	Others	Saccharides	up
D-Sucrose	C_6_H_10_O_7_	Others	Saccharides	up
D-Glucoronic acid	C_6_H_12_O_6_	Others	Saccharides	up
D-Mannose	C_6_H_12_O_6_	Others	Saccharides	up
D-Galactose	C_16_H_16_O_7_	Phenolic acids	Phenolic acids	up
Trans-5-O-(p-Coumaroyl) shikimate	C_16_H_18_O_8_	Phenolic acids	Phenolic acids	up

It is noteworthy that, in our results, all seven secondary metabolites were found to accumulate in TP (**Figure 6**; **Tables 3**, **4**). With the exception of D-Glucurono-6,3-lactone is saccharides and associated with the ascorbate and aldarate metabolism (ko00053) pathway, the remaining six compounds are involved in the flavonoid biosynthesis (ko00941) pathway (**Tables 3**, **4**). Further analysis of the six compounds involved in the flavonoid synthesis pathway which accumulated in TP revealed that: pinobanksin (3,5,7-trihydroxyflavanone) and aromadendrin (dihydrokaempferol) belong to the flavanonols subclass; naringenin chalcone (2’,4,4’,6’-Tetrahydroxychalcone) is classified as a chalcone; and naringenin (5,7,4’-Trihydroxyflavanone) is one of the flavanones and participates in the isoflavonoid biosynthesis (ko00943) pathway. Additionally, two phenolic acids, 5-O-p-coumaroylquinic acid and trans-5-O-(p-coumaroyl) shikimate, are implicated in the phenylpropanoid biosynthesis (ko00940) and stilbenoid, diarylheptanoid, and gingerol biosynthesis (ko00945) pathways (**Figures 4**, **6**; **Tables 3**, **4**).

### 3.4 Impact of four identified primary differential metabolites and rhoifolin on TT graft compatibility

In this study, enrichment analysis of metabolic pathways revealed that carbohydrate metabolic pathways constituted three of the eight enriched pathways, representing a relatively high proportion. Further investigation indicated that most metabolites within these pathways were primary metabolites accumulated in the scion leaves. To investigate whether the differential metabolites identified in the tomato leaves of scions grafted onto incompatible pepper rootstocks are indeed associated with graft compatibility, four TP-accumulated primary metabolites (glucose, mannose, sucrose, and trehalose) were selected for additional validation. These metabolites were exogenously applied to the scion cut surface before grafting to analyze their effects on the survival of compatible TT grafting at 5DAG by bend test. The results showed that, regarding primary metabolites, the external application of 20 mM and 50 mM glucose significantly increased the break of TT grafts (p< 0.001) (**Figure 8A**). Similarly, the external administration of 2.5% and 5% sucrose markedly increased the break of TT grafts (p< 0.001) (**Figure 8B**). For mannose, concentrations of 0.5 g/L and 1 g/L externally applied significantly increased the break of TT grafts (p< 0.01) (**Figure 8C**). In the case of trehalose treatment, the break percentage of TT grafts was 12.67% at an external concentration of 150 mM (p< 0.001), 29% at 300 mM, and rise to 44.14% at 600 mM (**Figure 8D**).

In addition to the 31 enriched metabolites, a substantial number of metabolites in the leaves of TP scions exhibited significant differences. Among these, the metabolite with the highest accumulation fold change was Apigenin-7-O-(2’’-O-apiosyl)(6’’-Malonyl)glucoside. Within the 168 accumulated metabolites, two Apigenin-like metabolites were also identified: Apigenin-7-O-rutinoside-4’-O-rhamnoside and Apigenin-7-O-neohesperidoside(Rhoifolin). Furthermore, among the 43 decreased metabolites, one Apigenin-like metabolite, Apigenin-4’-O-glucoside, was detected. These findings suggest that Apigenin-like metabolites may potentially influence graft compatibility. Consequently, we selected rhoifolin for further analysis of its role in the graft compatibility of TT. The results showed that as the concentration of exogenous rhoifolin increased (25, 50, 100 μM), the break rate of TT grafted seedlings at 5 DAG significantly increased (p< 0.001) (**Figure 8E**).

The results collectively demonstrated that these compounds significantly influence the survival of compatible TT grafts. The accumulated metabolites may participate in metabolic pathways associated with graft incompatibility and serve as potential biomarker candidates in scion leaves for identifying incompatible grafting combinations in this specific graft combination.

## 4 Discussion

### 4.1 The primary metabolites in the scion leaves may serve as potential markers for incompatible grafting identification

Elevated soluble sugars and starch have been reported at incompatible graft interfaces in grape and citrus (Loupit et al., 2022; He et al., 2022). In the present study, metabolomic analysis of TP scion leaves revealed that three of the eight significantly enriched pathways—Galactose metabolism (ko00052), Ascorbate and aldarate metabolism (ko00053), and Starch and sucrose metabolism (ko00500), were directly related to carbohydrate metabolism. Of the 17 enriched metabolites, 16 were upregulated, with 15 being primary metabolites including sucrose, glucose, mannose, galactose, trehalose, and trehalose-6-phosphate (T6P), collectively representing ~10% of all elevated metabolites. Additionally, N-methyl-GABA significantly accumulated in TP leaves, whereas proline was elevated but not statistically significant (p = 0.25), and histidine showed significant accumulation alongside decreased aspartic acid. Given that GABA and proline are known to accumulate under oxidative stress (Suzuki and Mittler, 2006), these primary metabolites may serve as characteristic indicators for early detection of delayed graft incompatibility.

These findings raise the intriguing hypothesis that carbohydrate accumulation in TP scion leaves reflects disrupted T6P–SnRK1 sugar signaling. T6P, synthesized by trehalose-6-phosphate synthase (TPS), integrates sugar metabolism and energy status via the SnRK1 kinase pathway, promoting anabolic processes essential for graft union formation (Baena-González and Lunn, 2020; Eh et al., 2024). We hypothesize that under incompatible grafting conditions, this signaling axis is disrupted, creating a localized sink deficiency at the graft junction that results in systemic sugar accumulation in scion leaves. The dose-dependent negative effect of exogenous trehalose on TT graft survival (12.67% at 150 mM, 29% at 300 mM, and 44.14% at 600 mM) supports the hypothesis that excessive sugar accumulation actively impairs graft union formation. Pharmacological or genetic manipulation of the T6P–SnRK1 pathway may represent a promising strategy to improve carbohydrate allocation, though future experimental validation is required to confirm this proposed mechanism.

### 4.2 Secondary metabolites involved in whole flavonoid biosynthesis pathway may serve as potential markers for incompatible grafting identification

The accumulation of polyphenolic compounds at graft interfaces has long been associated with incompatibility (Gebhardt and Feucht, 1982; Errea, 1998; Hou et al., 2023; Irisarri et al., 2016). Of the 207 differential metabolites identified in this study, 139 were secondary metabolites, of which 118, including 38 flavonoids and 18 phenolic acids, were accumulated in TP scion leaves. Notably, six of the seven metabolites associated with the eight significantly enriched KEGG pathways mapped to flavonoid biosynthesis (ko00941). Key pathway intermediates 5-O-p-coumaroylquinic acid and trans-5-O-(p-coumaroyl) shikimate, together with pinobanksin, aromadendrin, naringenin chalcone, and naringenin, were detected in TP leaves. Although ferulic acid itself was decreased in TP leaves, contradicting its reported role as an incompatibility marker in olive and grape (Azimi et al., 2016; Assunção et al., 2016), the accumulation of numerous glycosylated ferulic acid derivatives suggests complex tissue-specific dynamics of phenolic metabolism.

Although only six of the 31 enriched-pathway metabolites were flavonoids, flavonoids constituted the largest category among all 207 differential metabolites. Importantly, rhoifolin, an Apigenin glycoside that accumulated in TP leaves but was not among the enriched-pathway metabolites, significantly impaired TT graft compatibility. These results indicate that pathway-level evaluation of flavonoid metabolism offers greater diagnostic value than analysis of individual metabolites alone.

These findings raise the intriguing hypothesis that flavonoid and phenolic accumulation impairs graft compatibility through interference with cell wall remodeling. Graft union formation depends on cell wall loosening mediated by expansins, pectinases, and β-1,4-glucanases such as GH9B3 (Notaguchi et al., 2020). It is plausible that flavonoids and phenolic compounds inhibit these enzymes through protein oxidation or metal chelation (Sun et al., 2025), and that phenolic oxidative cross-linking with cell wall polysaccharides leads to cell wall rigidification rather than the loosening required for callus proliferation and vascular reconnection. In grapevine and Prunus, similar phenolic accumulations have been associated with dense, lignin-like barriers at incompatible interfaces (Zhang et al., 2022). These findings raise the intriguing hypothesis that the phenylpropanoid–flavonoid metabolic axis in scion leaves may serve as a systemic signaling hub modulating cell wall plasticity at the graft interface. Nevertheless, future experimental validation is required to confirm these proposed mechanisms.

### 4.3 Metabolic signatures in scion leaves are consistent with an immune-driven incompatibility mechanism

A recent study by Thomas et al. (2024) demonstrated that delayed tomato/pepper graft incompatibility is driven by an autoimmune-like response involving NLR upregulation, sustained defense hormone signaling (salicylic acid, jasmonic acid, ethylene), and localized programmed cell death (PCD) at the graft junction, accompanied by upregulated steroidal glycoalkaloid biosynthesis. Our metabolomic findings provide a metabolic counterpart to these observations: the substantial accumulation of flavonoids and phenolic acids in TP scion leaves aligns with phenylpropanoid pathway activation, a well-established branch of plant innate immunity. It is plausible that the elevated carbohydrate levels reflect immune-associated metabolic reprogramming and source–sink disruption, with impaired vascular reconnection further exacerbating sugar accumulation. Collectively, the concomitant accumulation of defensive secondary metabolites and primary metabolites in scion leaves suggests that scion leaf metabolomics offers a non-invasive window into the graft junction’s immune status, complementing transcriptomic insights from interface tissue.

It is important to emphasize that the mechanistic interpretations presented here remain speculative. The proposed roles of T6P–SnRK1 signaling disruption, flavonoid-mediated cell wall rigidification, and immune-driven metabolic reprogramming are inferred from correlative metabolomic data and require rigorous functional validation. Future experimental validation is required to confirm these hypotheses through transcriptomic profiling of key pathway genes, CRISPR-based gene editing of TPS, SnRK1, and phenylpropanoid pathway enzymes, and pharmacological manipulation of signaling pathways using specific agonists and inhibitors.

### 4.4 Study limitations and future perspectives

Several limitations should be acknowledged. First, the discovery-phase sample size (n = 6 per group) precluded formal ROC analysis. Although the OPLS-DA model showed excellent predictive capacity (Q² = 0.916), future studies should expand to ≥ 30 biological replicates per group and perform independent prospective validation to determine sensitivity, specificity, and AUC values. Second, the metabolic signatures were derived from a single scion/rootstock combination (tomato Zhongshu No. 4/pepper Hangjiao No. 1). Validation across multiple cultivars and other Solanaceous species is required to assess whether these metabolites represent universal markers or genotype-specific responses. Third, the single sampling time point (5 DAG) limits our understanding of the temporal dynamics of metabolic perturbations. Multi-time-point sampling (e.g., 3, 5, 7, 10, and 15 DAG) is essential to determine whether these changes are transient early responses or sustained pre-symptomatic indicators, and to establish the optimal diagnostic window.

In conclusion, scion leaves represent a viable, non-invasive material for early identification of delayed graft incompatibility. Among 207 differential metabolites, flavonoids were the most abundant category, and carbohydrate metabolism accounted for the largest proportion of enriched pathways. Exogenous validation confirmed that both carbohydrates (glucose, sucrose, mannose, trehalose) and flavonoids (rhoifolin) significantly impair graft compatibility. These findings suggest that pathway-level metabolic analysis offers a more comprehensive diagnostic perspective than individual metabolite profiling, though predictive utility requires further ROC-based validation and independent dataset testing.

## Data Availability

The original contributions presented in the study are included in the article/**Supplementary Material**, further inquiries can be directed to the corresponding author/s.
