# Supplementary material for: Identification of early metabolic indicator candidates in scion leaves associated with delayed incompatibility in tomato and pepper grafting via metabolism
Source: Front Plant Sci. 2026 Sep 1;17:1919357. doi: 10.3389/fpls.2026.1919357 (PMC13574693; doi:10.3389/fpls.2026.1919357)
Supplement: Supplementary file 1 [file DataSheet1.pdf]

Supplementary Material

Supplementary Figures

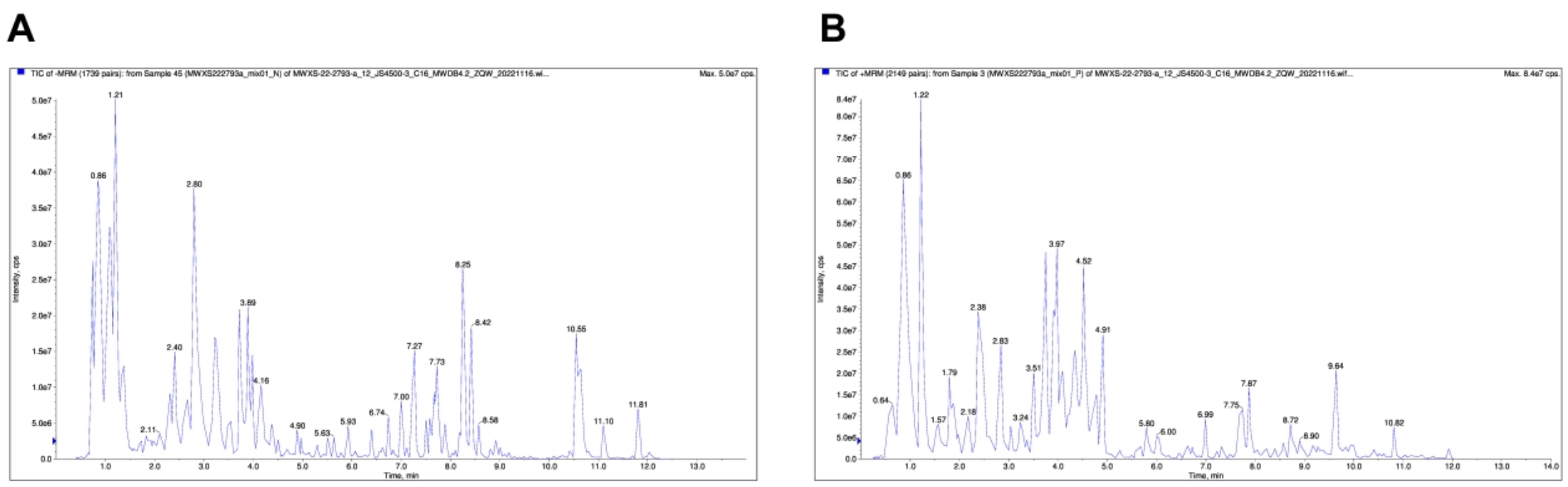

Supplementary Figure 1. Total Ion Chromatogram from Mass Spectrometry Analysis of Pooled Samples. A. N indicates the negative ion mode. B. P indicates the positive ion mode.

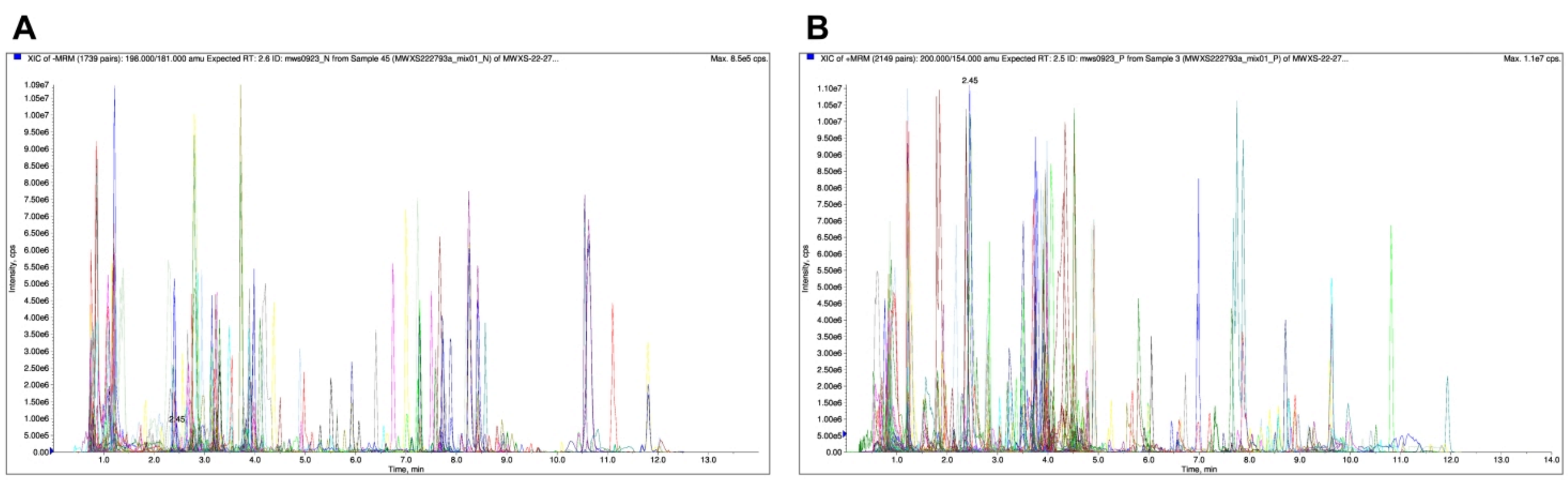

Supplementary Figure 2. Multi-Reaction Monitoring (MRM) Metabolite Detection with Multi-Peak Visualization . A. N indicates the negative ion mode. B. P indicates the positive ion mode.

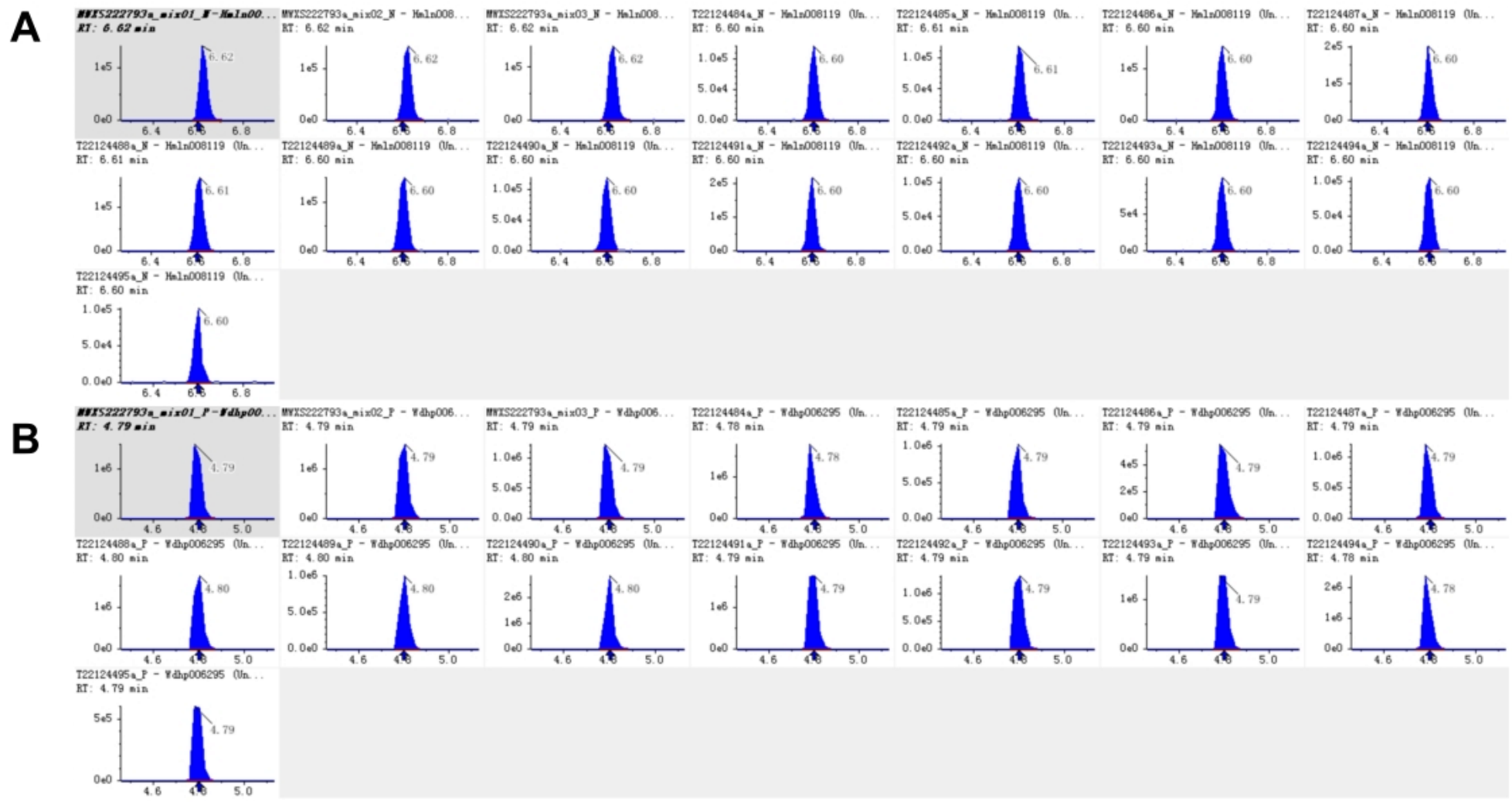

Supplementary Figure 3. Calibration Curve for Quantitative Analysis of Metabolites via Integration.

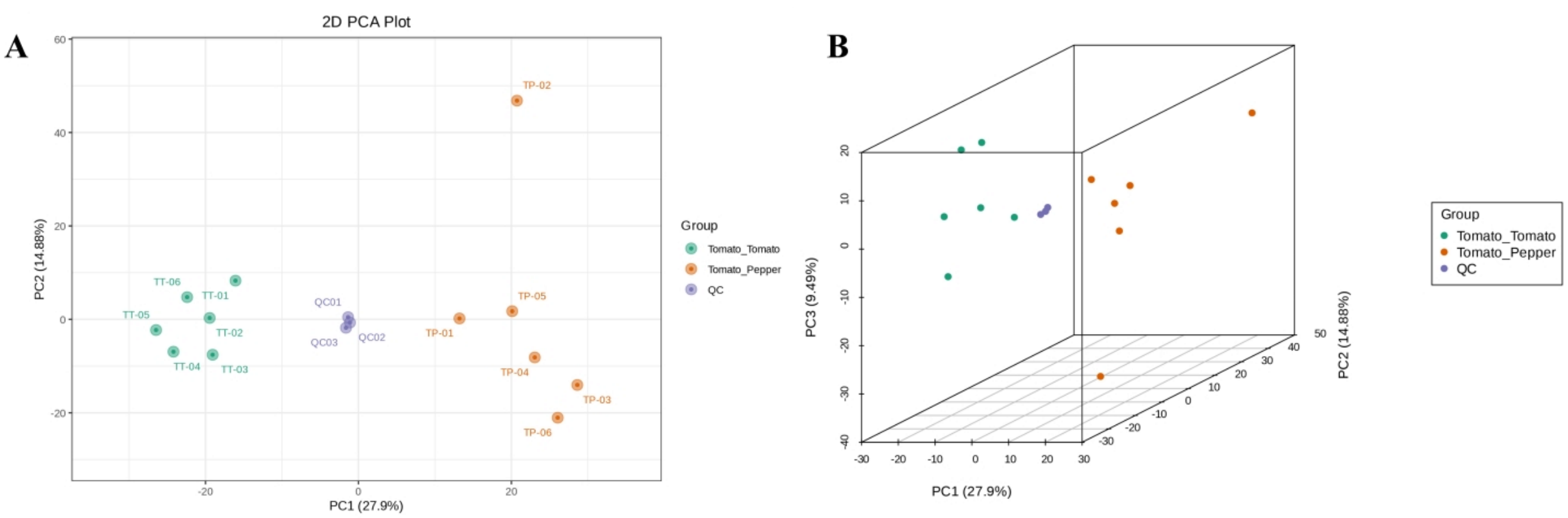

Supplementary Figure 4. The score plots of PCA for the mass spectrometry data of each sample group and the quality control samples. A. 2D score plot of PCA. B. 3D score of PCA.

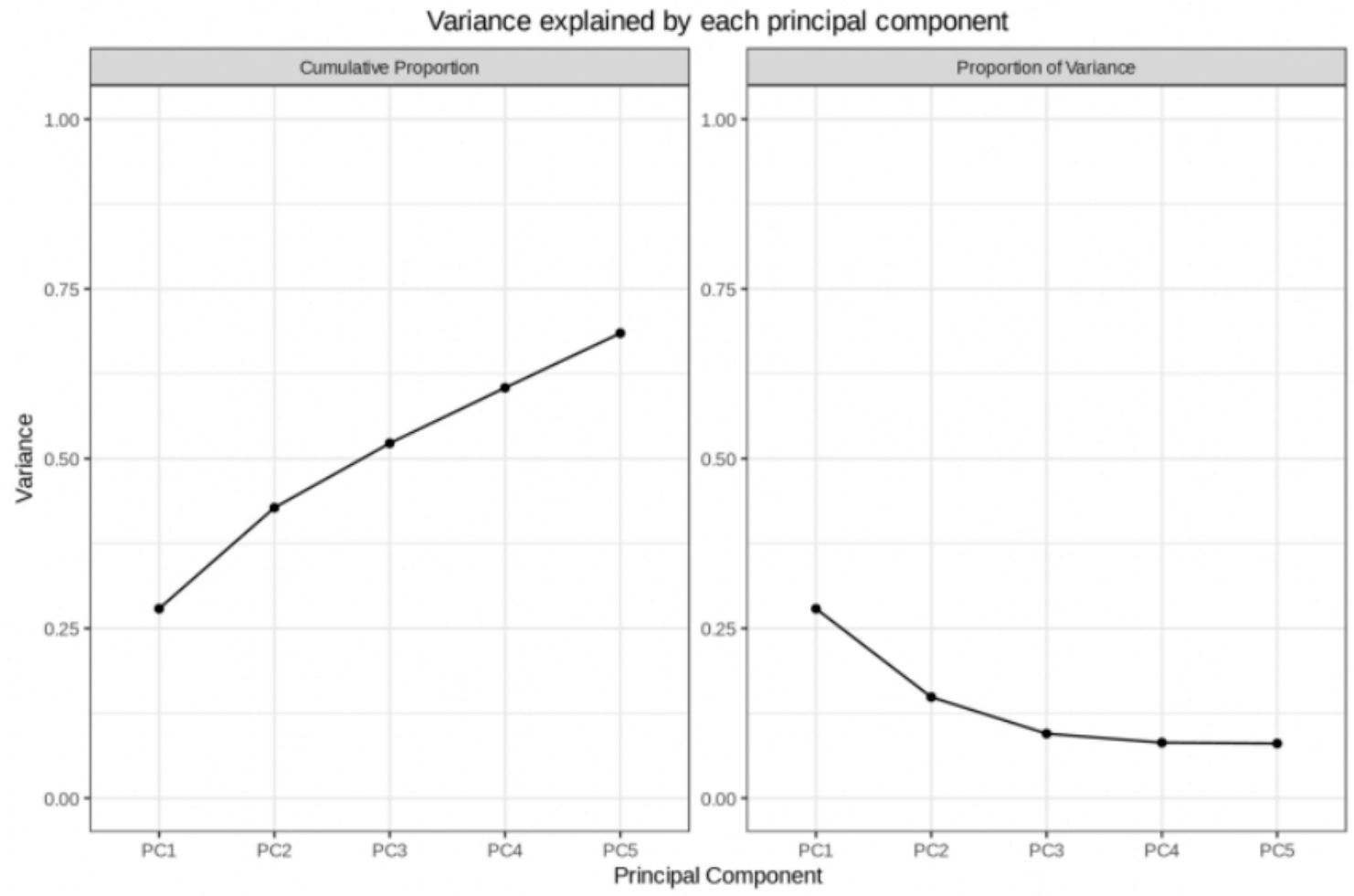

Supplementary Figure 5. The plot depicting the explanation rates of the first five principal components prior to PCA for the overall sample.

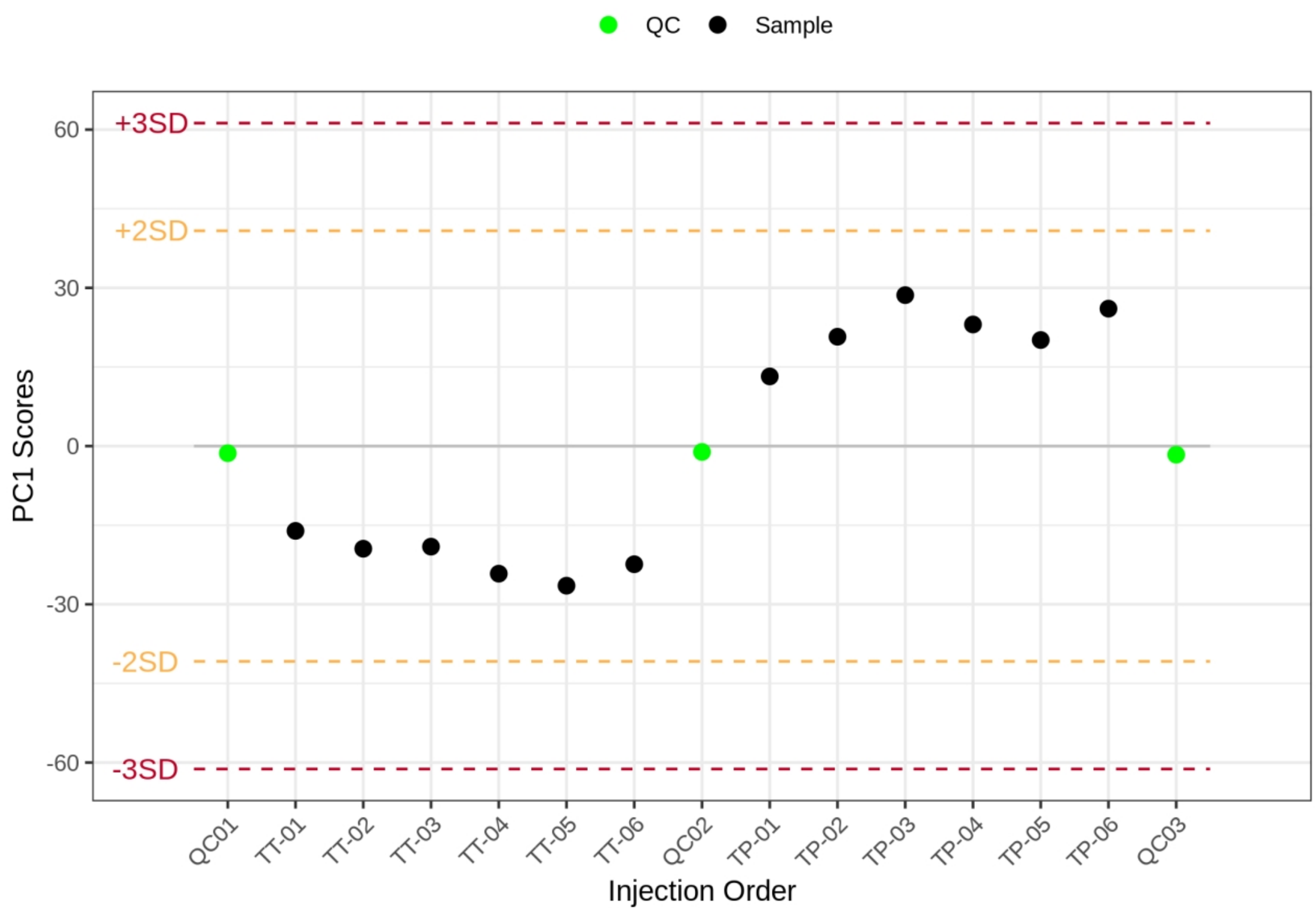

Supplementary Figure 6. Overall Sample Principal Component 1 (PC1) Control.

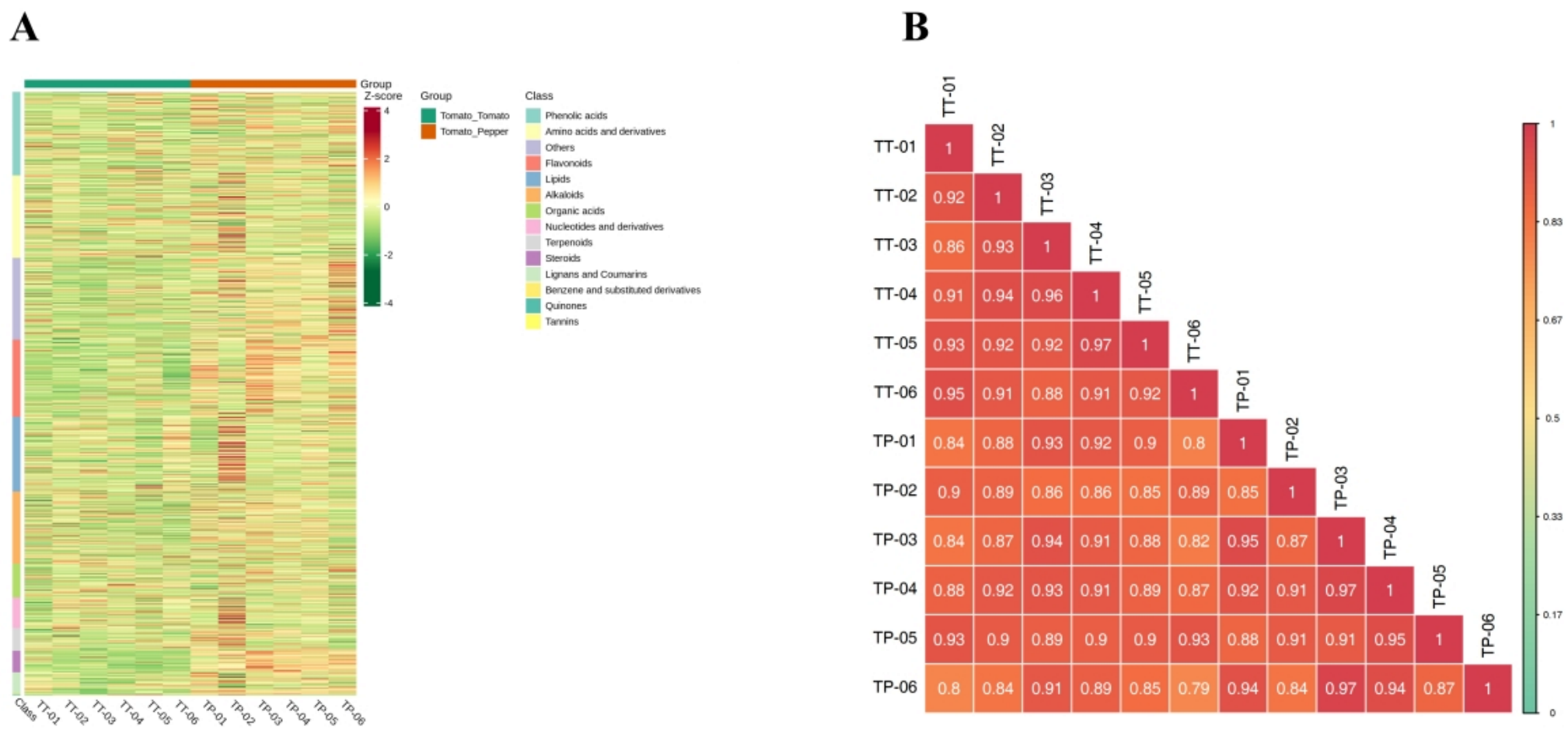

Supplementary Figure 7. Overall sample clustering plot (A) and Correlation Analysis of Samples (B).

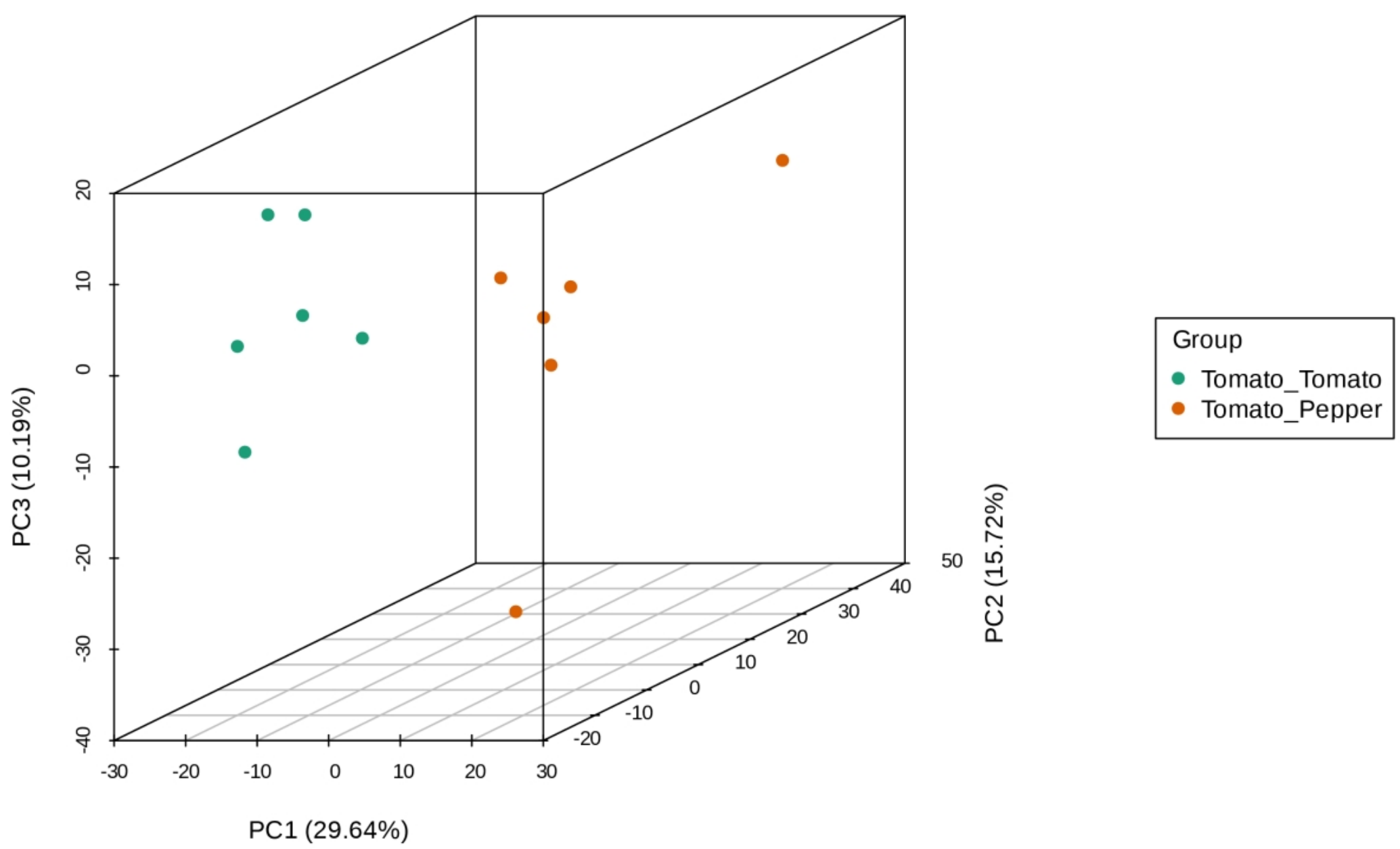

Supplementary Figure 8. 3D PCA among sample groups

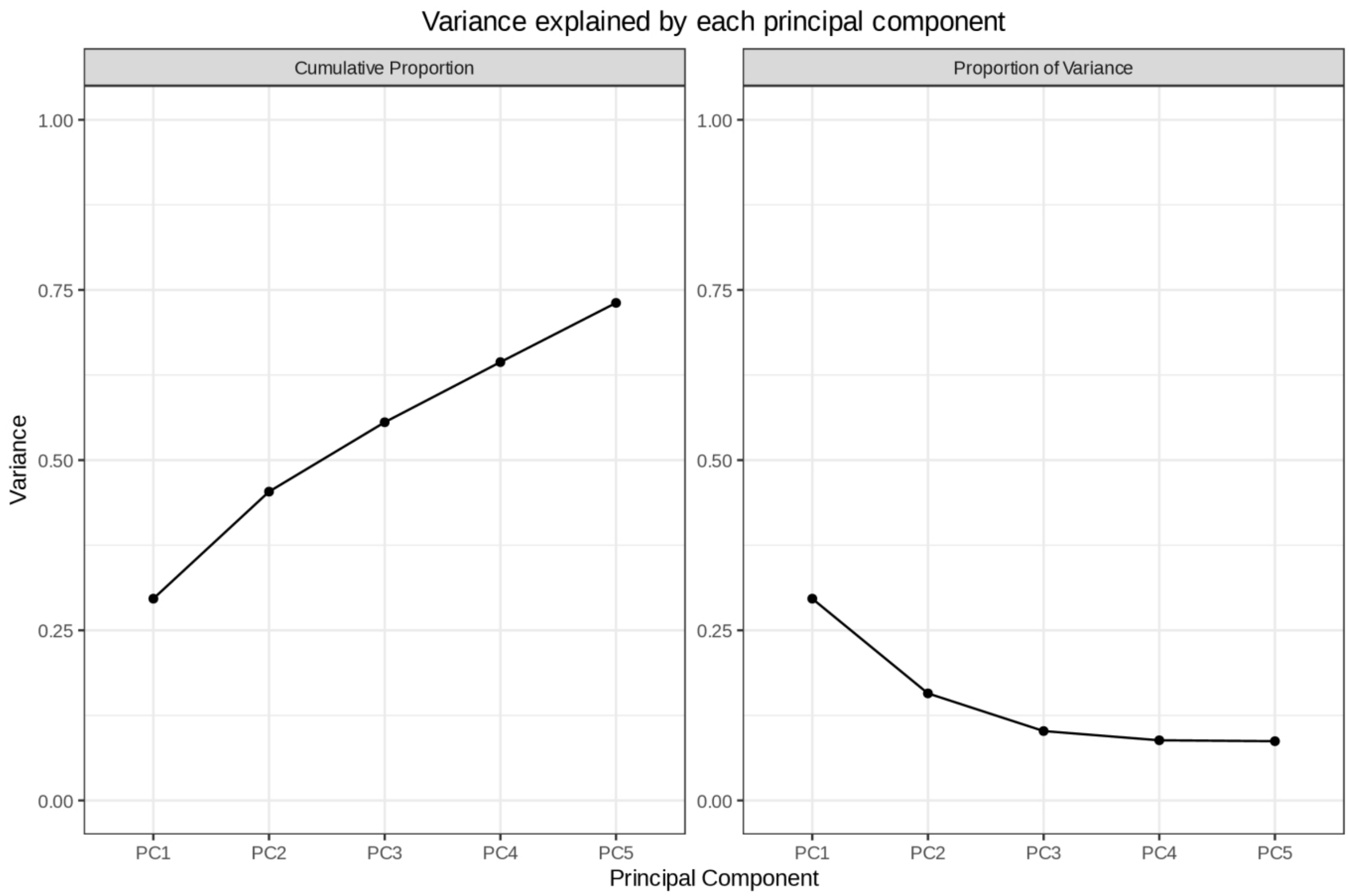

Supplementary Figure 9. Explained Variance Ratio in Group Principal Component Analysis.

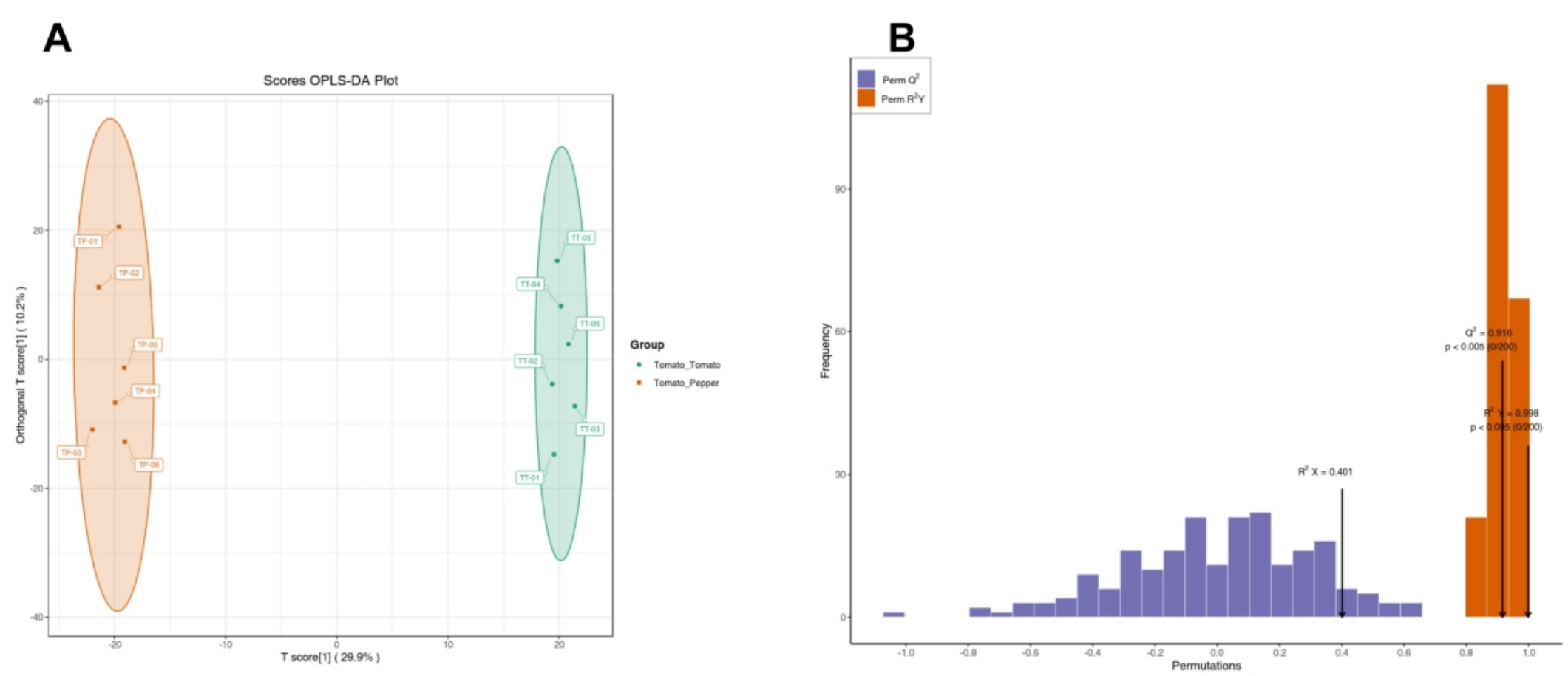

Supplementary Figure 10. Grouped OPLS-DA score plots and model validation diagrams. A. Score OPLS-DA plot. B. model validation diagrams.

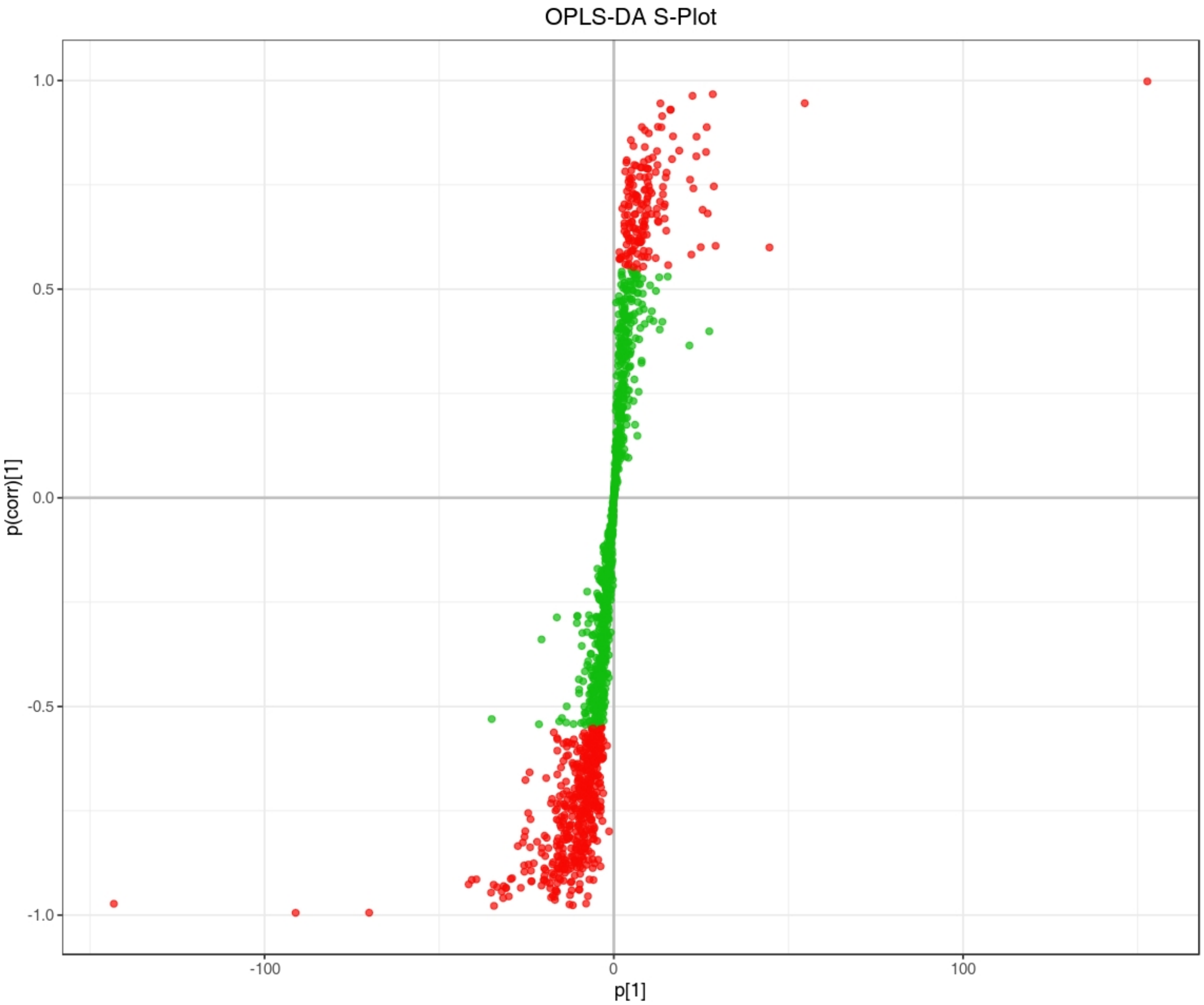

Figure Supplementary Figure 11. Group-wise OPLS-DA S-plot Analysis
